# Targeting PHGDH reverses the immunosuppressive phenotype of tumor-associated macrophages through α-ketoglutarate and mTORC1 signaling

**DOI:** 10.1038/s41423-024-01134-0

**Published:** 2024-02-27

**Authors:** Zhengnan Cai, Wan Li, Sonja Hager, Jayne Louise Wilson, Leila Afjehi-Sadat, Elke H. Heiss, Thomas Weichhart, Petra Heffeter, Wolfram Weckwerth

**Affiliations:** 1 Molecular Systems Biology (MOSYS), Department of Functional and Evolutionary Ecology, University of Vienna, Vienna, Austria; 2 Vienna Doctoral School of Ecology and Evolution, University of Vienna, Vienna, Austria; 3 Center for Cancer Research and Comprehensive Cancer Center, Medical University of Vienna, Vienna, Austria; 4 Department of Food Chemistry and Toxicology, Faculty of Chemistry, University of Vienna, Vienna, Austria; 5 Center for Pathobiochemistry and Genetics, Institute of Medical Genetics, Medical University of Vienna, Vienna, Austria; 6 Research Support Facility, Mass Spectrometry Unit, Faculty of Life Science, University of Vienna, Vienna, Austria; 7 Department of Pharmaceutical Sciences, University of Vienna, Vienna, Austria; 8 Vienna Metabolomics Center (VIME), University of Vienna, Vienna, Austria

**Keywords:** PHGDH, de novo serine synthesis, α-ketoglutarate, mTORC1, protumorigenic, tumor-associated macrophages, metabolomics, Tumour immunology, Cancer microenvironment

## Abstract

Phosphoglycerate dehydrogenase (PHGDH) has emerged as a crucial factor in macromolecule synthesis, neutralizing oxidative stress, and regulating methylation reactions in cancer cells, lymphocytes, and endothelial cells. However, the role of PHGDH in tumor-associated macrophages (TAMs) is poorly understood. Here, we found that the T helper 2 (Th2) cytokine interleukin-4 and tumor-conditioned media upregulate the expression of PHGDH in macrophages and promote immunosuppressive M2 macrophage activation and proliferation. Loss of PHGDH disrupts cellular metabolism and mitochondrial respiration, which are essential for immunosuppressive macrophages. Mechanistically, PHGDH-mediated serine biosynthesis promotes α-ketoglutarate production, which activates mTORC1 signaling and contributes to the maintenance of an M2-like macrophage phenotype in the tumor microenvironment. Genetic ablation of PHGDH in macrophages from tumor-bearing mice results in attenuated tumor growth, reduced TAM infiltration, a phenotypic shift of M2-like TAMs toward an M1-like phenotype, downregulated PD-L1 expression and enhanced antitumor T-cell immunity. Our study provides a strong basis for further exploration of PHGDH as a potential target to counteract TAM-mediated immunosuppression and hinder tumor progression.

## Introduction

Macrophages are key innate immune cells that play multiple roles in the maintenance of tissue homeostasis and exhibit phenotypic versatility in response to diverse tissue environments [1, 2]. The tumor microenvironment (TME) consists of malignantly transformed cells, stromal cells and infiltrating immune cells [3]. Tumor-associated macrophages (TAMs) are among the most abundant immune cells in the TME. These cells originate from circulating monocytes and tissue-resident macrophages that infiltrate tumor sites and undergo differentiation in response to multiple cytokines and growth factors (such as CCL2, CXCL4 and CSF-1) [4]. In most solid tumors, TAMs are regarded as overall protumorigenic factors that favor tumor growth and metastasis by promoting immunosuppression, angiogenesis and resistance to drug therapy and are generally correlated with a poor prognosis [5]. Several approaches involving the depletion of TAMs (with anti-CSF-1 antibodies and CSF-1R inhibition) [6, 7], the inhibition of immune checkpoints (with anti-PD-L1 antibodies) [8] and the reprogramming of TAMs (with agonistic anti-CD40 or inhibitory anti-CD47 antibodies) [9, 10] have been successful. However, a significant proportion of patients exhibit a limited response [11]. Further development of novel agents and strategies targeting additional immune checkpoints that benefit a wider range of patients is essential.

The proper activation and polarization of macrophages into distinct subtypes, although oversimplified, including M1-like and M2-like macrophage subsets, is crucial for their functional diversity [12]. Inflammatory (antitumorigenic) M1-like macrophages are commonly triggered by T helper 1 (Th1) cytokines, such as interferon gamma (IFN-γ) and tumor necrosis factor alpha (TNF-α), which are secreted by Th1 lymphocytes or lipopolysaccharide (LPS) from microbes. These cells secrete certain proinflammatory cytokines, including interleukin-6 (IL-6), IL-12, IL-1β and TNF-α, and produce inflammatory mediators, such as nitric oxide (NO) and reactive oxygen species (ROS), to activate antitumor immunity at the early stages of tumor onset [13]. Conversely, immunosuppressive (protumorigenic) M2-like macrophages are activated by Th2 cytokines, including IL-4, IL-13, and IL-10, which are secreted by the dominant Th2 lymphocytes in the TME or tumor cells at later stages, resulting in the upregulated expression of specific chemokines such as CCL17 and CCL22 and immunosuppressive cytokines such as IL-10 and transforming growth factor-β (TGF-β) [14]. Moreover, M2-like macrophages express high levels of certain markers, including ARG1, FIZZ1, YM1, MGL1, MGL2, CD36 and CD163 [15]. The changes in TAM phenotype during tumor progression reveals their crucial roles in tumor development and highlights the potential of targeting TAM infiltration, proliferation, and polarization as promising strategies for cancer immunotherapy.

We previously showed that IL-4-induced M2 macrophages activate phosphoglycerate dehydrogenase (PHGDH), which is the first rate-limiting enzyme of the serine synthesis pathway (SSP) and is required for their anti-inflammatory functions [16]. Furthermore, PHGDH was found to be highly expressed in TAMs isolated from murine orthotopic lung tumors [17], and its inhibition suppressed IL-4-stimulated M2 polarization and macrophage proliferation [16, 18]. These studies imply that PHGDH could be exploited for targeting TAMs and strengthening antitumor immunity. However, the influence of PHGDH on macrophage polarization has not been thoroughly defined and remains controversial. A previous study reported that LPS stimulation upregulates *Phgdh* expression as well as the ensuing one-carbon metabolism in bone marrow-derived macrophages (BMDMs), contributing to M1 polarization [19]. In contrast, another group found that LPS has no effect on the expression of *Phgdh* or other enzymes within the SSP in vitro, but exogenous serine is necessary for proinflammatory IL-1β production [20]. In addition, most experiments examining the effects of PHGDH mutation on tumor growth have utilized mouse strains lacking adaptive immunity, and systemic administration of pharmacological drugs concurrently affects both tumor cells and the TME. Thus, distinguishing the specific contribution of PHGDH to TAM-mediated effects on tumor development is challenging.

In this study, we applied genetic approaches to ablate the *Phgdh* gene in macrophages and discovered that PHGDH was necessary for the immunosuppressive activation of the Th2 cytokine IL-4. Deletion of *Phgdh* augmented the LPS-stimulated inflammatory phenotype. In a syngeneic mouse model of mesothelioma, we found that PHGDH expression was strongly increased in TAMs and that conditional deletion of *Phgdh* suppressed TAM infiltration and delayed tumor growth. Additionally, our findings suggest that the PHGDH-mediated SSP plays a crucial role in regulating the conversion of glutamate to alpha-ketoglutarate (αKG), which is necessary for mTORC1 signaling activation and sustains immunosuppressive M2 activation and TAM expansion.

## Results

### PHGDH supports macrophage immunosuppression

**Fig. 1:**
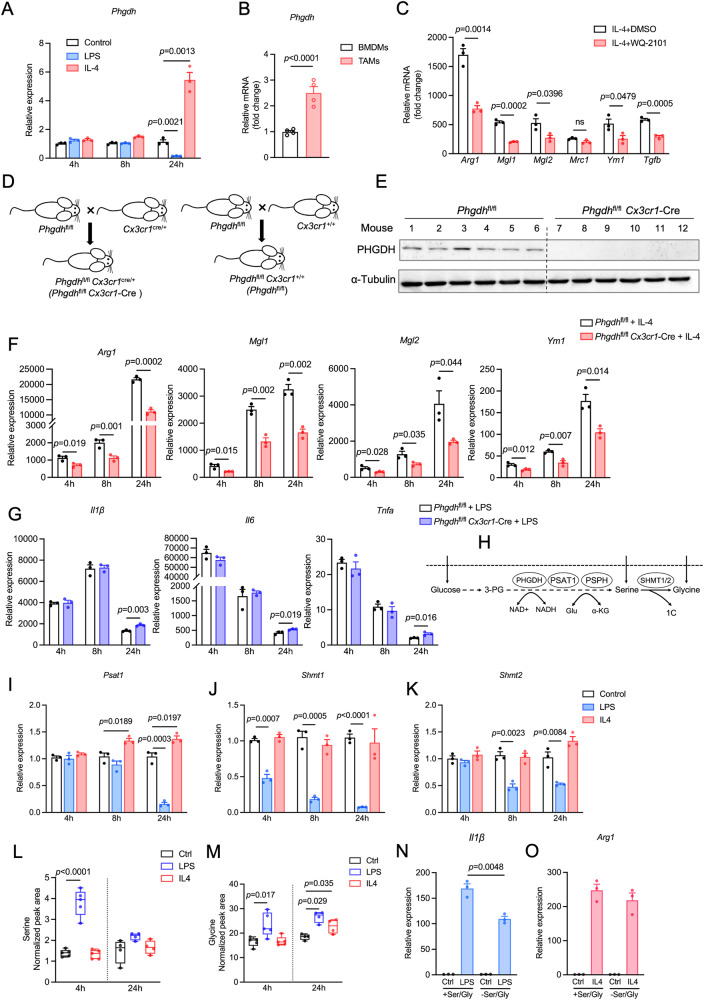
PHGDH deficiency suppresses IL-4-induced M2 polarization but exacerbates LPS-induced proinflammatory activation. **A** qPCR analysis of *Phgdh* mRNA in murine wild-type (WT) BMDMs incubated with DMEM (control), IL-4 (20 ng/mL), or LPS (100 ng/mL) for 4 h (hours), 8 h and 24 h (*n* = 3 independent experiments). **B** qPCR analysis of *Phgdh* mRNA in TAMs (CD11b^+^F4/80^+^) sorted from AE17 mesothelioma cells and BMDMs (control) from nontumor-bearing mice (*n* = 4 biologically independent samples). **C** qPCR analysis of M2 markers in IL-4-induced BMDMs treated with dimethyl sulfoxide (DMSO) or 25 μM WQ-2101 for 24 h (*n* = 3 independent experiments). The data are expressed as the fold change relative to the control (DMEM-treated WT BMDMs). **D** Generation of macrophage-specific *Phgdh* knockout mice (*Phgdh*^fl/fl^ *Cx3cr1*-Cre) by crossing *Phgdh*^fl/fl^ mice with mice expressing the Cre recombinase under the control of the Cx3cr1 promotor (*Cx3cr1*^cre/+^) and control mice (*Phgdh*^fl/fl^) by crossing *Phgdh*^fl/fl^ mice with *Cx3cr1*^+/+^ mice. **E** Western blot analysis of PHGDH protein expression in BMDMs from PHGDH-deficient mice (*Phgdh*^fl/fl^ *Cx3cr1*-Cre) and control mice (*Phgdh*^fl/fl^). **F** qPCR analysis of M2 markers in IL-4-treated PHGDH-deficient BMDMs and IL-4-stimulated *Phgdh*^fl/fl^ BMDMs after 4, 8 and 24 h (*n* = 3 biologically independent samples). The data are expressed as the fold change relative to the control (DMEM-treated *Phgdh*^fl/fl^ BMDMs). **G** qPCR analysis of M1 markers in LPS-stimulated PHGDH-deficient BMDMs and LPS-stimulated *Phgdh*^fl/fl^ BMDMs after 4 h, 8 h, and 24 h (*n* = 3 biologically independent samples). The data are expressed as the fold change relative to the control (DMEM-treated *Phgdh*^fl/fl^ BMDMs). **H** Schematic of serine metabolism, including de novo serine synthesis and one-carbon units. qPCR analysis of *Psat1* (**I**), *Shmt1* (**J**) and *Shmt2* (**K**) in wild-type (WT) BMDMs incubated with DMEM (control), IL-4 (20 ng/mL), or LPS (100 ng/mL) for 4 h, 8 h or 24 h (*n* = 3 independent experiments). Intracellular levels of serine (**L**) and glycine (**M**) in extracts of BMDMs stimulated with IL-4 (20 ng/mL), LPS (100 ng/mL) or DMEM (control) for 4 h and 24 h (*n* = 4 independent experiments). MS peak areas were normalized to internal standards and corresponding pellet protein concentrations. **N**, **O** qPCR analysis of *Il1β* (**M**) and *Arg1* (**N**) in wild-type (WT) BMDMs stimulated with LPS (100 ng/mL) or IL-4 (20 ng/mL) for 4 h in complete DMEM or in complete serine and glycine-depleted (−SG) media (*n* = 3 independent experiments). The data are shown as the mean ± SEM. Statistical significance was calculated using a two-tailed unpaired Student’s *t* test

Gene expression changes following stimulation are highly dynamic, and both gene repression and activation occur at specific time points [21]. Therefore, we stimulated BMDMs with IL-4 or LPS for 3 different periods of time (4 h or 8 h for the early immune response, and 24 h for the late immune response) to investigate the temporal dynamics of gene expression changes involved in macrophage polarization and SSP. Additionally, we evaluated TAMs from mice bearing AE17 mesothelioma tumors (Supplementary Fig. S1A). We found upregulated expression of the PHGDH protein and gene in IL-4-treated macrophages (M2) at 24 h (Fig. 1A and Supplementary Fig. S1B) and upregulated expression of the *Phgdh* messenger RNA (mRNA) in TAMs compared with BMDMs (Fig. 1B). In contrast, LPS stimulation (in M1 macrophages) did not change *Phgdh* mRNA expression at early time points (4 h and 8 h) (Fig. 1A) but did significantly downregulate *Phgdh* gene expression at 24 h. Treatment with the selective PHGDH inhibitor PKUMDL-WQ-2101 (WQ-2101) significantly inhibited the IL-4-induced M2 markers *Arg1*, *Mgl1*, *Mgl2*, *Ym1* and *Tgfb* (Fig. 1C). Notably, these effects of PHGDH inhibition were observed in media supplemented with normal levels of exogenous serine, suggesting that factors other than serine production may play a crucial role in IL-4-induced M2 macrophage polarization. To further study the intrinsic effect of PHGDH on macrophages, we generated myeloid-specific Phgdh knockout mice by crossing *Phgdh*^fl/fl^ mice with *Cx3cr1*^cre/+^ mice (*Phgdh*^fl/fl^ *Cx3cr1*^cre/+^; denoted as *Phgdh*^fl/fl^ *Cx3cr1*-Cre) and control mice by crossing *Phgdh*^fl/fl^ mice with *Cx3cr1*^+/+^ mice (*Phgdh*^fl/fl^ *Cx3cr1*^+/+^; denoted as *Phgdh*^fl/fl^) (Fig. 1D). We confirmed the knockout of PHGDH in macrophages by Western blot analysis of BMDMs from *Phgdh*^fl/fl^ *Cx3cr1*-Cre mice compared to those from *Phgdh*^fl/fl^ mice (Fig. 1E). We observed that the conditional deletion of *Phgdh* did not affect the viability of naive macrophages (Supplementary Fig. S1C), yet M2 polarization was significantly restrained in PHGDH-deficient macrophages upon IL-4 stimulation over time (Fig. 1F). In contrast, PHGDH deficiency had no effect on the expression of the proinflammatory genes *Il1β*, *Il6* and *Tnfα* at 4 h or 8 h (Fig. 1G) but did upregulate *Il1β*, *Il6* and *Tnfα* following LPS treatment at 24 h (Fig. 1G). Thus, these results indicate that PHGDH activity promotes an immunosuppressive M2 phenotype in macrophages.

PHGDH is the first and rate-limiting enzyme of SSP (Fig. 1H). We next asked whether serine metabolism contributes to PHGDH-mediated macrophage polarization. Like that of *Phgdh*, phosphoserine transaminase 1 (*Psat1*) mRNA expression was significantly downregulated upon LPS stimulation but modestly increased upon IL-4 treatment at 24 h (Fig. 1I). Cytoplasmic *Shmt1* expression was strongly downregulated over time upon exposure to LPS, and mitochondrial *Shmt2* was also reduced beginning at 8 h after LPS exposure (Fig. 1J, K). However, IL-4 stimulation had no impact on the expression of these genes (Fig. 1J, K). Genetic deletion of *Phgdh* had no obvious effect on these genes following stimulation with IL-4 or LPS (Supplementary Fig. S1D, E). Moreover, cellular serine and glycine levels were significantly increased upon LPS stimulation (Fig. 1L, M), suggesting that LPS may enhance the import of exogenous serine and glycine to maintain the demand of M1 macrophages. IL-4 stimulation had no effect on serine or glycine levels at 4 h but modestly elevated glycine levels at 24 h (Fig. 1L, M). The alanine-serine-cysteine-threonine transporters ASCT1 and ASCT2, encoded by *Slc1a4* and *Slc1a5*, respectively, have been reported to mediate serine and glycine transport [22]. We therefore measured the gene expression of *Slc1a4* and *Slc1a5* upon LPS or IL-4 stimulation. Surprisingly, the mRNA expression of *Slc1a4* was not affected by LPS stimulation at 4 or 24 h (Supplementary Fig. S1F), whereas *Slc1a4* was upregulated by IL-4 stimulation at 4 h (Supplementary Fig. S1F). *Slc1a5* expression was even downregulated upon LPS stimulation but was not affected by IL-4 stimulation (Supplementary Fig. S1J). We then proceeded to investigate whether exogenous serine and glycine levels influence macrophage polarization. Inhibition of ASCTs by the specific inhibitor L-phenylglycine (l-phg) [23] downregulated LPS-induced *Il1β* expression (Supplementary Fig. S1H) but not IL-4-induced *Arg1 expression* (Supplementary Fig. S1I). Furthermore, the absence of serine and glycine (-SG) in the medium reduced LPS-induced *Il1β* (Fig. 1N), whereas the IL-4-induced M2 marker *Arg1* (Fig. 1O) was not affected by conditional deprivation, highlighting the importance of exogenous serine and glycine for LPS-induced M1 polarization [19] but not for IL4-induced M2 polarization [16]. Taken together, these findings indicate that PHGDH is necessary for IL-4-induced immunosuppressive macrophage activation but is dispensable for the LPS-stimulated inflammatory phenotype.

### PHGDH contributes to M2-like TEM polarization and macrophage proliferation

**Fig. 2:**
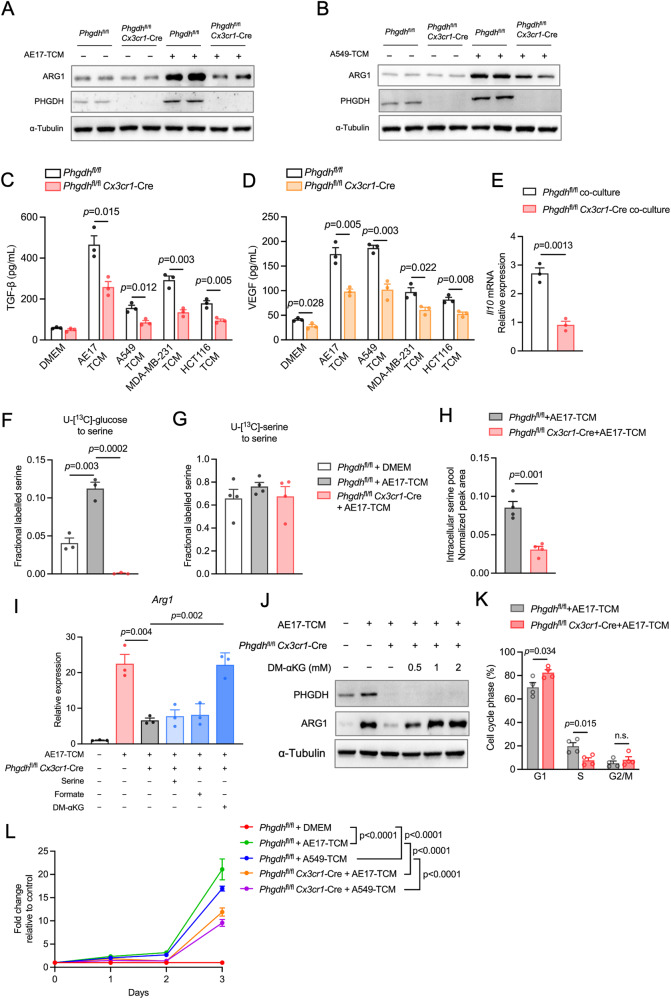
PHGDH-mediated de novo serine synthesis supports M2-like TEM polarization and macrophage proliferation. Western blot detection of ARG1 and PHGDH in *Phgdh*^fl/fl^ *Cx3cr1*-Cre BMDMs and *Phgdh*^fl/fl^ BMDMs incubated with DMEM, AE17-TCM (**A**) or A549-TCM (**B**) for 24 h. Protein secretion of TGF-β (**C**) and VEGF (**D**) in PHGDH-deficient BMDMs and *Phgdh*^fl/fl^ BMDMs incubated with AE17-TCM, A549-TCM, MDA-MB-231-TCM or HCT116-TCM for 24 h (*n* = 3 biologically independent samples). **E** qPCR analysis of *Il-10* in PHGDH-deficient BMDMs and control BMDMs cocultured with AE17 cells for 24 h (*n* = 3 independent experiments). The data are expressed as the fold change relative to the control (*Phgdh*^fl/fl^ BMDMs cocultured with DMEM). **F** ^13^C-labeling of serine in PHGDH-deficient BMDMs and *Phgdh*^fl/fl^ BMDMs incubated with AE17-TCM for 24 h and then pulsed with U-[^13^C]-glucose for 2 h (*n* = 3 independent experiments). **G** ^13^C-labeling of serine in PHGDH-deficient BMDMs and *Phgdh*^fl/fl^ BMDMs incubated with AE17-TCM for 24 h and then pulsed with U-[^13^C]-serine for 2 h (*n* = 3 independent experiments). **H** Intracellular level of serine in control BMDMs and PHGDH-deficient BMDMs (*n* = 4 independent experiments). **I** qPCR analysis of *Arg1* expression in PHGDH-deficient BMDMs incubated with AE17-TCM supplemented with serine, formate or 1 mM dimethyl-α-ketoglutarate (DM-αKG) (*n* = 3 independent experiments). **J** Western blot analysis of PHGDH and ARG1 in PHGDH-deficient BMDMs incubated with AE17-TCM supplemented with 0.5 mM, 1 mM, 2 mM or without DM-αKG. **K** Cell cycle analysis of BMDMs from PHGDH-deficient and control mice by 7-AAD and BrdU staining (*n* = 4 independent experiments). n.s. nonsignificant. **L** PHGDH-deficient BMDMs and control BMDMs were treated with AE17-TCM or A549-TCM for the indicated days and then incubated for 1 h with CCK8 reagent (*n* = 4 biologically independent samples). The optical density (OD) was detected at 450 nm. The data were normalized to the control (DMEM-treated *Phgdh*^fl/fl^ BMDMs) and are expressed as the fold change relative to the control. All the data are shown as the mean ± SEM. Statistical significance was calculated using a two-tailed unpaired Student’s *t* test

Given the highly M2-like nature of TAMs in the complex TME and the high expression of *Phgdh* in isolated TAMs, we investigated whether PHGDH is required for the function of TAMs. We generated tumor-educated macrophages (TEMs) by culturing *Phgdh*^fl/fl^ and PHGDH-deficient BMDMs in tumor-conditioned media (TCM) derived from cancer cell lines with high PHGDH amplification; these cells included murine AE17 mesothelioma, human A549 lung cancer, human HCT116 colon cancer, and human MDA-MB-231 breast cancer cells without significant PHGDH amplification (Supplementary Fig. S2A). We found that all of the activated *Phgdh*^fl/fl^ TEMs exhibited upregulated expression of the ARG1 protein (Fig. 2A, B, Supplementary Fig. S2B, C) and the release of the immunosuppressive cytokine transforming growth factor-β (TGF-β) (Fig. 2C). However, deletion of *Phgdh* downregulated the expression of ARG1 and TGF-β (Fig. 2A–C, Supplementary Fig. S2B, C). The expression of another TAM marker, vascular endothelial growth factor (VEGF; also called VEGF-A), was also reduced by PHGDH deficiency (Fig. 2D), suggesting that PHGDH plays a crucial role in promoting M2-like TEM polarization, regardless of the PHGDH levels in the corresponding cancer cells. Moreover, coculture of *Phgdh*^fl/fl^ BMDMs with AE17 cells further upregulated the expression of the anti-inflammatory cytokine *Il10* in response to LPS, but PHGDH deficiency downregulated the expression of *Il10* (Fig. 2E).

We next sought to evaluate whether serine metabolism was necessary for M2 macrophage polarization in the tumor context. Notably, serine levels were decreased in AE17-TCM, A549-TCM and MDA-MB-231-TCM cells, while glycine levels were increased compared to those in fresh or macrophage-conditioned medium (BMDM-CM) (Supplementary Fig. S2D, E). After incubation, the extracellular levels of both serine and glycine remained unchanged in the TEMs (Supplementary Fig. S2D, E), suggesting that exogenous serine is not necessary for TEM activation. To verify this, we performed pulsed stable isotope labeling with uniformly ^13^C-labeled (U-[^13^C]) glucose or serine, followed by gas chromatography‒mass spectrometry (GC‒MS) analysis. We found that, compared with the control, AE17-TCM-induced TEMs significantly increased the incorporation of glucose-derived carbons into serine (Fig. 2F), whereas the uptake of U-[^13^C]-serine was comparable between the TEMs and the control (Fig. 2G). Notably, PHGDH deficiency markedly reduced the incorporation of labeled glucose into serine (Fig. 2F) and decreased the intracellular serine pool (Fig. 2H). However, the demand of TEMs for exogenous serine was not affected by PHGDH deficiency (Fig. 2G). Therefore, TEMs rely on PHGDH-mediated de novo serine synthesis to maintain their intracellular serine pools.

We then asked whether supplementation with key metabolites of serine metabolism could restore the immunosuppressive phenotype in PHGDH-deficient TEMs. Surprisingly, serine and formate (known to be a one-carbon donor) were unable to restore M2-like activation in the TEMs (Fig. 2I). However, supplementation with cell-permeable αKG, the byproduct of SSP (Fig. 1H), restored the mRNA and protein expression of the M2 marker ARG1 in PHGDH-deficient TEMs (Fig. 2I, J). Importantly, we observed a concentration-dependent effect of αKG on ARG1 expression (Fig. 2J). In addition, both the AE17-TCM- and A549-TCM-treated BMDMs exhibited greater confluence and elongated morphology than the untreated BMDMs (Supplementary Fig. S2F). PHGDH deficiency led to a reduced cell number and altered morphology (Supplementary Fig. S2F) but did not induce apoptosis, as evidenced by the unchanged cleaved (cl.) caspase-3 (Supplementary Fig. S2G). Cell cycle analysis revealed that the cell cycle progression of PHGDH-deficient macrophages was inhibited, as indicated by a decreased percentage of cells in S-phase and increased percentage of cells in G1-phase (Fig. 2K). We next performed a CCK8 assay to evaluate the effects of PHGDH on TEM proliferation. As BMDMs differentiate into primary macrophages with limited proliferative capacity, we observed low OD450 values in both TEMs and untreated BMDMs (control) (Supplementary Fig. S2H). However, the increasing relative OD450 values of the AE17-TCM- and A549-TCM-treated TEMs indicated that the proliferative capacity of the TEMs was greater than that of the control (Fig. 2L). In contrast, genetic deletion of *Phgdh* suppressed the proliferation of TEMs (Fig. 2L). Taken together, these results indicate that PHGDH-mediated de novo serine synthesis is essential for M2-like TEM polarization and macrophage proliferation.

### PHGDH suppression impairs TAM infiltration and tumor growth

**Fig. 3:**
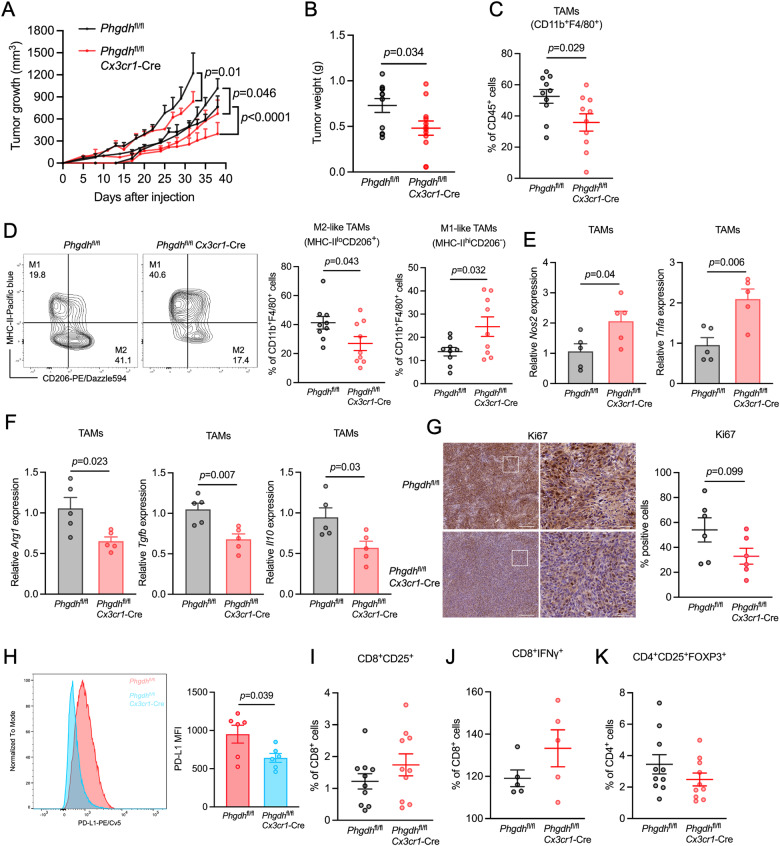
Macrophage-specific *Phgdh* ablation reduces tumor growth, TAM infiltration and polarization. **A** Tumor growth curves showing the mean tumor volume at the indicated timepoints following implantation of AE17 cells into *Phgdh*^fl/fl^ (batch1, *n* = 5; batch2, *n* = 3; batch3, *n* = 3) and *Phgdh*^fl/fl^ *Cx3cr1*-Cre (batch1, *n* = 5; batch2, *n* = 3; batch3, *n* = 4) mice. The data represent three independent experiments. **B** Tumor weights of mice injected with AE17 mesothelioma cells from **A**. The tumor data were collected on Day 32, Day 35 or Day 38 after tumor transplantation (*Phgdh*^fl/fl^, *n* = 11 in total; *Phgdh*^fl/fl^ *Cx3cr1*-Cre, *n* = 12 in total). Each symbol represents one individual. **C** Flow cytometry analysis of the proportion of TAMs (CD45^+^CD11b^+^F4/80^+^) among tumors from *Phgdh*^fl/fl^ (*n* = 10) and *Phgdh*^fl/fl^ *Cx3cr1*-Cre mice (*n* = 10). The data represent three independent experiments. **D** Flow cytometry analysis of M1-like TAMs (MHC-II^hi^CD206^−^) and M2-like TAMs (MHC-II^lo^CD206^+^) from *Phgdh*^fl/fl^ tumors (*n* = 9) and *Phgdh*^fl/fl^ *Cx3cr1*-Cre tumors (*n* = 9). The data represent three independent experiments. qPCR analysis of the antitumorigenic markers *Nos2* and *Tnfa* (**E**) and the protumorigenic markers *Arg1*, *Tgfb*, and *Il10* (**F**) in TAMs sorted from mice with AE17 tumors (*n* = 5 tumors per condition). The data are expressed as the fold change relative to the control. The results represent two independent experiments. **G** Representative immunohistochemical (IHC) images (left) and quantification (right) of Ki67-stained cells in tumor sections from mice with AE17 mesothelioma tumors (*n* = 6 tumors per condition). The data represent two independent experiments. Scale bar, 200 µm (left), 50 µm (right). **H** Representative histogram (left) and quantitative plot (right) of PD-L1^+^ TAMs in tumor sections from mice with AE17 tumors (*n* = 6 tumors per condition). The data represent two independent experiments. Analysis of the proportions of activated CD8^+^ (CD3^+^CD8^+^CD25^+^) (**I**, *n* = 10 tumors per condition), CD8^+^IFNγ^+^ (**J**, *n* = 5 tumors per condition) and Treg (CD4^+^CD25^+^FOXP3^+^) (**K**, *n* = 10 tumors per condition) cells from mice with AE17 mesothelioma tumors. The results represent three independent experiments (**I** and **K**) or two independent experiments (**J**). All of the data are shown as the mean ± SEM. Statistical significance was calculated using two-way ANOVA with Tukey’s multiple comparison test (**A**) or a two-tailed unpaired Student’s *t* test (**B**–**K**)

We hypothesized that the loss of PHGDH in macrophages, which results in attenuated M2-like TEM polarization and proliferation, may confer greater antitumor immunity in vivo. To test this hypothesis, we subcutaneously injected *Phgdh*^fl/fl^ and *Phgdh*^fl/fl^ *Cx3cr1*-Cre mice with AE17 cells and monitored tumor growth over time. In three independent experiments, we consistently found that both tumor volume (Fig. 3A and Supplementary Fig. S3A) and tumor weight were lower (by 34%, *p* = 0.034) (Fig. 3B) in the macrophage-specific *Phgdh*-knockout mice (8–12 weeks) than in the control mice (8–12 weeks) and corresponded to a significant reduction in TAM (CD45^+^CD11b^+^F4/80^+^) infiltration (Fig. 3C and Supplementary Fig. S3B). Moreover, depletion of PHGDH markedly decreased the population of M2-like TAMs (MHC-II^lo^CD206^+^) but increased the population of M1-like TAMs (MHC-II^hi^CD206^−^) (Fig. 3D and Supplementary Fig. S3B). To examine the influence of PHGDH ablation on the expression of macrophage-associated markers, we used fluorescence-activated cell sorting to isolate TAMs from *Phgdh*^fl/fl^ *Cx3cr1*-Cre tumor-bearing mice and analyzed the mRNA expression of several protumorigenic and proinflammatory markers. We found that compared with TAMs from *Phgdh*^fl/fl^ tumor-bearing mice, those from *Phgdh*^fl/fl^ *Cx3cr1*-Cre tumor-bearing mice exhibited significant upregulation of genes linked to an M1-like/proinflammatory phenotype (*Nos2* and *Tnfa*) (Fig. 3E), while downregulated genes were associated with an M2-like/protumorigenic phenotype (*Arg1*, *Tgfb* and *Il10*) (Fig. 3F). In addition to traditional M1-/M2-like TAMs, four distinct macrophage subsets have been characterized in human tumor tissues in a previous study, revealing the heterogeneity of macrophages in the TME [24]. To understand the impact of PHGDH on different cell populations, we selected 1–2 markers for each phenotype and conducted qPCR analysis. We found that PHGDH deficiency downregulated *Slc40a1* and *Gpnmb*, which are markers for the Mφ-c2-C1QA subset and *Apoe*, a marker for the Mφ-c3-APOE subset, while it had no effect on the other two subsets, Mφ-c1-THBS1 (represented by *Thbs1*) and Mφ-c4-GPX3 (represented by *Lst1* and *Fcgr3*) (Supplementary Fig. S3C). Taken together, these findings indicate that PHGDH selectively modulates the behavior of specific TAM subsets in the TME. In addition to traditional M2-like TAMs, Mφ-c2-C1QA and Mφ-c3-APOE TAMs are inhibited, indicating the crucial role of PHGDH in regulating these particular macrophage populations. In contrast, the results for the unaffected cell subsets, namely, Mφ-c1-THBS1 and Mφ-c4-GPX3, suggested that these cells were functionally independent of PHGDH or potentially regulated by alternative pathways. These findings suggest that targeting PHGDH in macrophages may reprogram certain TAM populations and decrease tumor malignancy. Indeed, the number of Ki67^+^ proliferating cells was slightly lower in *Phgdh*^fl/fl^ *Cx3cr1*-Cre tumors than in *Phgdh*^fl/fl^ tumors, although the difference did not reach statistical significance (Fig. 3G). Furthermore, we observed significant downregulation of PD-L1 in *Phgdh*^fl/fl^ *Cx3cr1*-Cre TAMs compared to *Phgdh*^fl/fl^ TAMs (Fig. 3H), suggesting that PHGDH deficiency may impact antitumor T-cell functions. Surprisingly, PHGDH deficiency had no impact on the total T-cell population or on the CD4 or CD8 subsets (Supplementary Figs. S3D and S4A). However, we observed a modest increase in CD8^+^CD25^+^ and CD8^+^IFNγ^+^ T cells (Fig. 3I, J), while regulatory T cells (CD4^+^FOXP3^+^CD25^+^ Tregs) were decreased (Fig. 3K). Earlier studies have reported that *Cx3cr1* is also expressed in lymphocytes [25]. To exclude the possibility of off-target effects of the *Cx3cr1*-Cre strain on the status of PHGDH in T cells, we evaluated PHGDH protein expression in T cells within tumor tissues via immunofluorescence staining of CD3. As shown in Supplementary Fig. S4B, there was no obvious difference in PHGDH expression in T cells between *Phgdh*^fl/fl^ and *Phgdh*^fl/fl^ *Cx3cr1*-Cre tumor-bearing mice. This finding suggested that the *Phgdh*^fl/fl^ *Cx3cr1*-Cre strain does not influence the expression of PHGDH in T cells. Notably, we conducted similar experiments in older mice (16–18 weeks) but observed no significant antitumor effects of PHGDH conditional ablation in these mice (data not shown), suggesting that the immune system of older mice might be less responsive to the alterations induced by PHGDH deficiency. Taken together, our findings indicate that the loss of PHGDH not only results in reduced TAM infiltration in younger mice and a restrained immunosuppressive phenotype but also leads to the inhibition of tumor growth and partial restoration of antitumor immunity.

### PHGDH suppression drives macrophage metabolic reprogramming

**Fig. 4:**
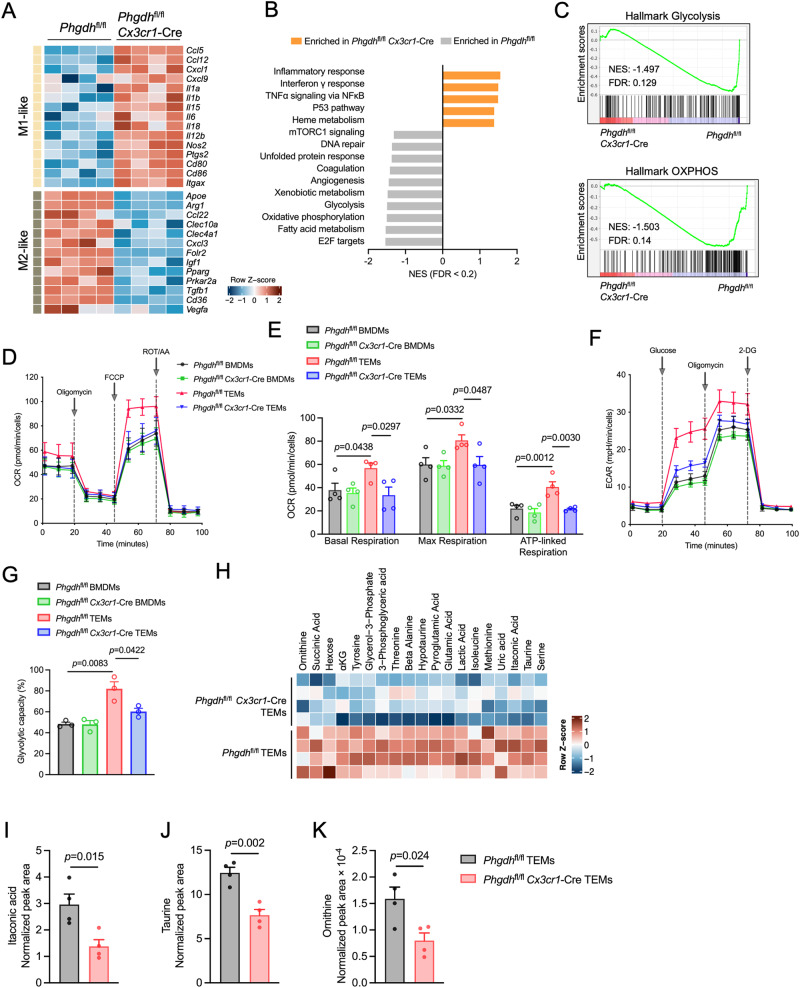
PHGDH deficiency modulates macrophage metabolic reprogramming. **A** Heatmap of M1 and M2 macrophage gene expression in *Phgdh*^fl/fl^ *Cx3cr1*-Cre and *Phgdh*^fl/fl^ BMDMs incubated with AE17-TCM for 24 h (*n*  =  4 biologically independent samples). **B** GSEA was conducted using hallmark gene sets from the Molecular Signatures Database of the Broad Institute. The analysis identified the most significantly enriched gene sets in *Phgdh*^fl/fl^ *Cx3cr1*-Cre and control BMDMs. **C** GSEA plots of the glycolysis (up) and oxidative phosphorylation (OXPHOS) (down) gene signatures in *Phgdh*^fl/fl^ *Cx3cr1*-Cre TEMs relative to *Phgdh*^fl/fl^ TEMs from the analysis in B with their respective normalized enrichment scores (NES) and false discovery rates (FDR). **D** Oxygen consumption rate (OCR) of *Phgdh*^fl/fl^ *Cx3cr1*-Cre BMDMs and control BMDMs incubated with AE17-TCM or DMEM for 24 h followed by exposure to the indicated bioenergetic modulators: oligomycin, carbonyl cyanide 4-(trifluoromethoxy) phenylhydrazone (FCCP), and rotenone/antimycin A (AA) (*n* = 4 independent experiments). The arrow denotes when the compounds were added. **E** Basal OCR, maximal OCR, and ATP-linked respiration from the trace in **A**. **F** Glycolysis stress analysis of *Phgdh*^fl/fl^ *Cx3cr1*-Cre BMDMs and control BMDMs incubated with AE17-TCM or DMEM for 24 h before exposure to the indicated compounds: glucose, oligomycin, and 2-deoxy-D-glucose (2-DG) (*n* = 4 independent experiments). The arrow denotes when the compounds were added. **G** Glycolytic capacity of the trace in **B**. **H** Cellular levels of significantly reduced metabolites in PHGDH-deficient BMDMs compared to those in control BMDMs incubated with AE17-TCM for 24 h (*n* = 4 biologically independent samples). The color bar indicates the range of metabolite levels depicted by the Z score. GC‒MS analysis of the cellular metabolites itaconic acid (**I**), taurine (**J**) and ornithine (**K**) (*n* = 4 biologically independent samples). MS peak areas were normalized to the internal standard and corresponding pellet protein concentration. The data are shown as the mean ± SEM. Statistical significance was calculated using a two-tailed unpaired Student’s *t* test

To shed light on the mechanisms by which PHGDH maintains the immunosuppressive phenotype of TAMs, we performed transcriptomic analyses of *Phgdh*^fl/fl^ and *Phgdh*^fl/fl^ *Cx3cr1*-Cre macrophages from AE17 cells treated with TCM. Notably, PHGDH deficiency not only suppressed the TCM-induced expression of genes associated with M2-like (protumorigenic) polarization, as indicated by the expression of *Arg1*, *Tgfb, Mgl1* (*Clec10a*), *Ccl22, and Cd36* but also strongly stimulated the expression of genes associated with an M1-like (antitumorigenic) signature (e.g., *Ccl5*, *Cxcl1*, *Cxcl9*, *Cd86*, *Il18* and *Nos2*) (Fig. 4A). Gene set enrichment analysis (GSEA) revealed that genetic ablation of PHGDH notably decreased the “E2F transcription factor target”, “mitochondrial oxidative phosphorylation” (OXPHOS), “glycolysis”, and “mTORC1 signaling” signatures and concurrently augmented the “inflammatory response” and “p53 pathway” (Fig. 4B, C, Supplementary Fig. S5A–C), implying that PHGDH plays a major role in regulating the cell cycle and metabolic reprogramming. KEGG enrichment analysis revealed dramatic downregulation of numerous metabolic pathways, including “pyrimidine metabolism”, “purine metabolism”, “arginine and proline metabolism”, and “glycine, serine and threonine metabolism”, in *Phgdh*^fl/fl^ *Cx3cr1*-Cre TEMs (Supplementary Fig. S5D), all of which are purportedly critical for supporting the function of M2 macrophages and cell proliferation. We then assessed whether PHGDH deficiency could cause metabolic reprogramming in TEMs. We measured the oxygen consumption rate (OCR), an indicator of OXPHOS, and the extracellular acidification rate (ECAR), a readout of glycolysis. We found that the basal OCR, maximal OCR and ATP-linked respiration of *Phgdh*^fl/fl^ TEMs were strongly elevated compared to those of the control (DMEM-treated *Phgdh*^fl/fl^ BMDMs) (Fig. 4D, E), suggesting an increase in mitochondrial OXPHOS. However, this increase was not observed in PHGDH-deficient cells (Fig. 4D, E), indicating that mitochondrial respiration was inhibited upon PHGDH depletion in TEMs. In addition, both DMEM-treated *Phgdh*^fl/fl^ BMDMs (control) and *Phgdh*^fl/fl^ TEMs cultured in the absence of glucose had similar ECARs (Fig. 4F), whereas the addition of glucose significantly increased the ECAR in *Phgdh*^fl/fl^ TEMs but not in the control (Fig. 4F), consistent with an increase in the Warburg effect in TAMs [26]. Indeed, the conversion of glucose to lactate represented approximately 50% of the maximal glycolytic ability of control macrophages, whereas *Phgdh*^fl/fl^ TEMs used up to 80% of their maximal ability (Fig. 4G). However, PHGDH deficiency significantly reduced the glycolytic capacity triggered by TCM (Fig. 4F, G). These data suggest that, in contrast to the lower overall metabolic activity observed in the control macrophages and PHGDH-deficient TEMs, the *Phgdh*^fl/fl^ TEMs were highly metabolically active. Consistent with these findings, cellular metabolite levels were distinctly different between *Phgdh*^fl/fl^ *Cx3cr1*-Cre TEMs and *Phgdh*^fl/fl^ TEMs (Supplementary Fig. S5E and Supplementary Table S1). In addition, we observed a significant decrease in the levels of the anti-inflammatory metabolites itaconate and taurine (Fig. 4H–J) as well as the polyamine metabolite ornithine (Fig. 4K), which are essential for immunosuppressive macrophage functions in PHGDH-deficient TEMs. These findings suggest that depletion of PHGDH shifts TEMs away from an immunosuppressive metabolic state.

To verify these results in vivo, we evaluated representative genes of the glycolytic pathway and the subunits of the five protein complexes involved in the electron transport chain (ETC) in TAMs sorted from *Phgdh*^fl/fl^ *Cx3cr1*-Cre and *Phgdh*^fl/fl^ tumor-bearing mice. We found that the expression of most representative genes (*Ndufb8*, *Sdhb*, *Cox5a* and *Atp5a1*) encoding for the ETC was lower in *Phgdh*^fl/fl^ *Cx3cr1*-Cre TAMs than in *Phgdh*^fl/fl^ TAMs (Supplementary Fig. S5F), indicating that PHGDH deficiency reduced TAM OXPHOS, which was consistent with our in vitro Seahorse analyses. Furthermore, compared with their *Phgdh*^fl/fl^ counterparts, *Phgdh*^fl/fl^ *Cx3cr1*-Cre TAMs exhibited a slight decrease (not significant) in the expression of selected genes (*Hk2*, *Gaphd*, *Pgam1*, *Pfkm*, *Pkm* and *Ldha*) involved in glycolysis (Supplementary Fig. S5G). This nuanced effect suggested the possibility of a compensatory response from increased M1-like TAM populations within the tumor tissues of *Phgdh*^fl/fl^ *Cx3cr1*-Cre mice. Moreover, we evaluated two other enzymes (*Psat1* and *Psph*) involved in the de novo serine synthesis pathway (Supplementary Fig. S5H). The *Psat1* and *Psph* gene expression levels did not differ between *Phgdh*^fl/fl^ *Cx3cr1*-Cre TAMs and *Phgdh*^fl/fl^ TAMs, which is consistent with our in vitro data (Supplementary Fig. S1D, E), indicating that the regulatory effect of PHGDH on the other two enzymes is minimal.

### De novo serine synthesis promotes αKG-dependent immunosuppressive macrophage activation

**Fig. 5:**
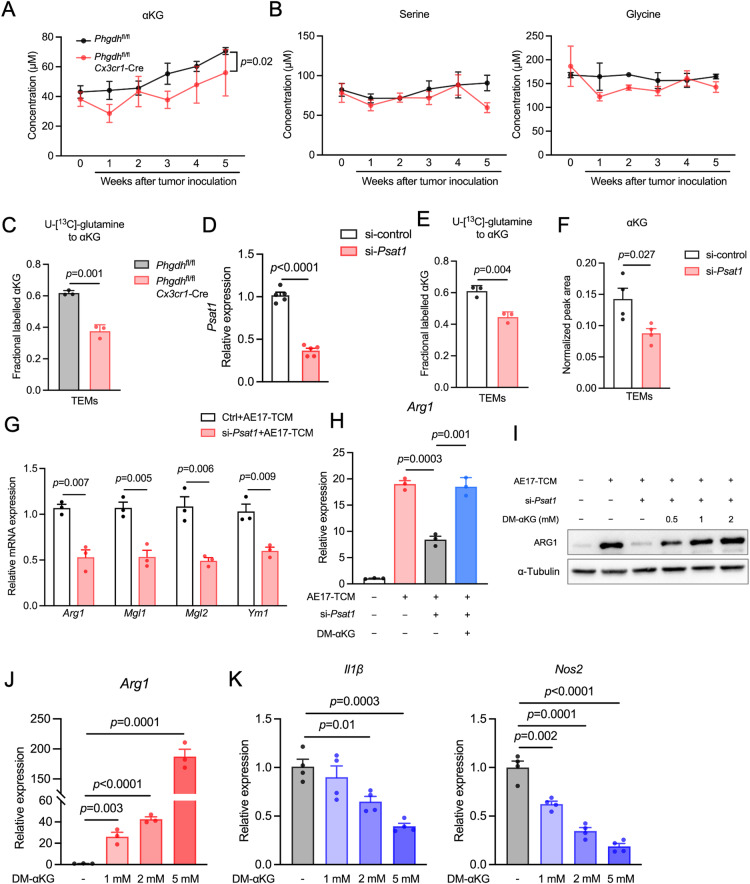
De novo serine synthesis regulates the conversion of glutamate to αKG, contributing to the maintenance of an M2-like macrophage phenotype. The concentrations of αKG (**A**), serine (**B**) and glycine (**B**) in the serum of control and PHGDH-deficient mice at the indicated days post tumor inoculation. (*n* = 4 biologically independent samples). **C** ^13^C-labeling of αKG in PHGDH-deficient BMDMs and *Phgdh*^fl/fl^ BMDMs incubated with AE17-TCM for 24 h and then pulsed with U-[^13^C]-glutamine for 2 h (*n* = 3 independent experiments). **D** qPCR analysis of *Psat1* gene expression in RAW 264.7 macrophages subjected to *Psat1* siRNA (si-*Psat1*) or nontargeting siRNA (si-control) treatment (*n* = 5 biologically independent samples). The data are expressed as the fold change relative to the control. **E** ^13^C-labeled αKG in si-*Psat1* RAW and si-control RAW264.7 macrophages incubated with AE17-TCM for 24 h and then pulsed with U-[^13^C]-glutamine for 2 h (*n* = 3 independent experiments). **F** Cellular level of αKG in si-*Psat1-* and si-control RAW 264.7 macrophages incubated with AE17-TCM for 24 h, followed by GC‒MS (*n* = 3 biologically independent samples). **G** qPCR analysis of the M2 markers *Arg1*, *Mgl1*, *Mgl2* and *Ym1* in *Psat1*-knockdown and control macrophages incubated with AE17-TCM for 24 h (*n* = 3 biologically independent samples). The data are expressed as the fold change relative to the level in the AE17-TCM-treated si-control group. **H** qPCR analysis of *Arg1* expression in Psat1-knockdown macrophages incubated with AE17-TCM supplemented with or without 1 mM DM-αKG (*n* = 3 independent experiments). **I** Western blot analysis of ARG1 in *Psat1*-knockdown macrophages incubated with AE17-TCM supplemented with (0.5 mM, 1 mM or 2 mM) or without DM-αKG. qPCR analysis of *Arg1* (**J**), *Il1β* (**K**) and *Nos2* (**K**) expression in *Psat1*-knockdown macrophages incubated with AE17-TCM supplemented with the indicated concentrations of DM-αKG (*n* = 3 biologically independent samples). The data are expressed as the fold change relative to the control. The data are shown as the mean ± SEM. All the data were analyzed using a two-tailed unpaired Student’s *t* test (**C**–**H**, **J**–**K**) or two-way ANOVA with Tukey’s multiple comparisons test (**B**)

Based on our metabolomic analysis, PHGDH depletion inhibited glutamine utilization in TEMs, as indicated by reduced intracellular levels of glutamate and αKG (Fig. 4H). Our in vivo data confirmed that the serum αKG levels in PHGDH conditional knockout mice were considerably decreased upon tumor injection (Fig. 5A). Interestingly, the serum levels of serine and glycine remained unaffected by the conditional knockout of PHGDH (Fig. 5B), indicating that the daily dietary intake of these nonessential amino acids provided an adequate supply. These findings suggest that the reduced TAM functions observed in PHGDH-deficient mice may not be attributed to alterations in serine and glycine availability. αKG is the key intermediate of the TCA cycle and can be directly linked *via* the de novo serine synthesis pathway, which produces equimolar amounts of serine and αKG because the enzyme PSAT1 converts 3-phosphohydroxypyruvate and glutamine-derived glutamate to phosphoserine and αKG (Fig. 1H) [27]. We hypothesized that SSP plays a crucial role in the conversion of glutamate to αKG and thereby promotes TAM polarization toward an M2-like phenotype. To address this hypothesis, we performed pulsed stable labeling studies using U-[^13^C]-glutamine and revealed that the carbon flow from glutamine to αKG was significantly lower in PHGDH-deficient TEMs (by 40%) than in control cells (Fig. 5C). We then utilized siRNA to silence the expression of *Psat1* in RAW264.7 cells (Fig. 5D). Like in the case of genetic ablation of *Phgdh*, siRNA-mediated *Psat1* suppression decreased U-[^13^C]-glutamine incorporation into αKG (Fig. 5E) and decreased the intracellular αKG level in TEMs (Fig. 5F). In addition, *Psat1* suppression blunted M2-like polarization compared with that in the control group (Fig. 5G). We then investigated whether rescuing the αKG level in *Psat1*-silenced TEMs could restore the immunosuppressive phenotype. As expected, the addition of αKG to AE17-TCM restored macrophage *Arg1* mRNA and protein expression in *Psat1*-silenced TEMs (Fig. 5H, I). Given that supplementation with αKG could restore M2-like TEM polarization, we then asked whether αKG treatment alone is capable of inducing M2-like polarization. Indeed, αKG treatment alone activated *Arg1* expression in a concentration-dependent manner (Fig. 5I–J). In contrast, the expression of the M1-like markers *Il1β* and *Nos2* was strongly inhibited upon αKG treatment (Fig. 5K). Taken together, these results indicate that de novo serine synthesis plays a crucial role in the conversion of glutamate to αKG, contributing to the maintenance of the M2-like phenotype.

### αKG generated by SSP supports mTORC1 signaling

**Fig. 6:**
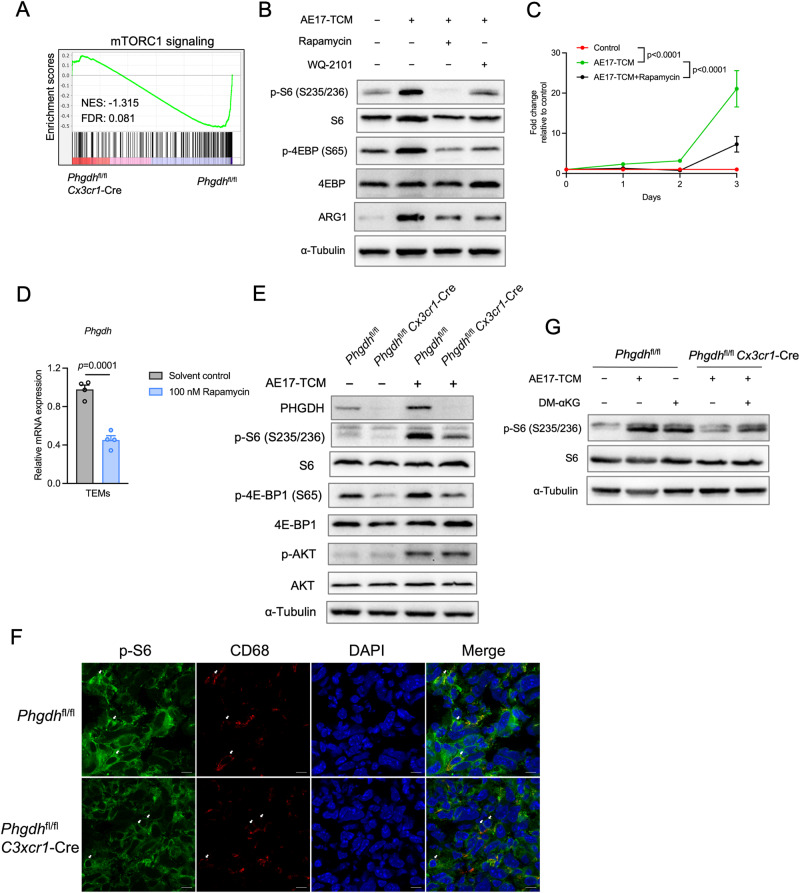
The PHGDH-mediated SSP activates mTORC1 signaling in M2-like TAMs. **A** GSEA plot of the “mTORC1 signaling” gene signature in *Phgdh*^fl/fl^ *Cx3cr1*-Cre TEMs relative to that in *Phgdh*^fl/fl^ TEMs from the analysis in Fig. 4B with the respective NES and FDR. **B** Western blot analysis of the indicated proteins in WT BMDMs incubated with AE17-TCM supplemented with 100 nM rapamycin or 25 μM WQ-2101 for 24 h. **C** *Phgdh*^fl/fl^ BMDMs were treated with AE17-TCM for the indicated days and then incubated for 1 h with CCK8 reagent (*n* = 4 biologically independent samples). The OD was detected at 450 nm. The data were normalized to the control (DMEM-treated *Phgdh*^fl/fl^ BMDMs) and are expressed as the fold change. **D** qPCR analysis of *Phgdh* expression in AE17-TCM-treated BMDMs treated with or without 100 nM rapamycin for 24 h (*n* = 4 biologically independent samples). **E** Western blot analysis of the indicated proteins in PHGDH-deficient BMDMs and control BMDMs incubated with or without AE17-TCM for 24 h. **F** Representative images of cryosectioned AE17 tumors stained with antibodies against p-S6 (green) and CD68 (red) as well as the nuclear stain DAPI. Scale bar, 10 μm. **G** Western blot analysis of the indicated proteins in PHGDH-deficient BMDMs and control BMDMs incubated with AE17-TCM supplemented with or without 1 mM DM-αKG for 24 h. The data are shown as the mean ± SD (**C**) or SEM (**D**). The data were analyzed using two-way ANOVA with Tukey’s multiple comparisons test (**C**) or a two-tailed unpaired Student’s *t* test (**D**)

Our GSEA data highlighted the enrichment of the mTORC1 signaling pathway in PHGDH-deficient TEMs (Fig. 6A and Supplementary Fig. S6A). Previous studies have shown that both serine and αKG can support mTORC1 signaling in cancer and immune cells [28–31], and our results underscore the regulatory effects of αKG produced by de novo serine synthesis on M2-like TEM polarization. We reasoned that mTORC1 is involved in TAM/TEM M2-like polarization and proliferation driven by de novo serine synthesis pathway-derived αKG. To evaluate mTORC1 signaling, we assessed the phosphorylation of the downstream effector S6 and eukaryotic translation initiation factor 4E-binding protein 1 (4E-BP1). We observed that, compared with control cells, both AE17-TCM- and A549-TCM-stimulated TEMs exhibited substantial S6 and 4EBP1 phosphorylation (Fig. 6B, Supplementary Fig. S6B–D). Rapamycin treatment not only blocked mTORC1 signaling activation (Fig. 6B, Supplementary Fig. S6B–D) but also suppressed TEM M2-like polarization (Fig. 6B and Supplementary Fig. S6C) and macrophage proliferation (Fig. 6C and Supplementary Fig. S6E). Notably, rapamycin treatment suppressed *Phgdh* mRNA expression in TEMs (Fig. 6D), implying that mTORC1 signaling and PHGDH interact with each other. Like rapamycin, WQ-2101 treatment reduced the phosphorylation of these mTORC1 targets (Fig. 6B, Supplementary Fig. S6B–D). Furthermore, genetic deletion of PHGDH diminished mTORC1 signaling in TEMs and in tumors (Fig. 6E, F). We also observed that the activity of mTORC2, as indicated by the phosphorylation of AKT, was induced by the tumor medium but was not affected by the ablation of PHGDH (Fig. 6E). Next, we asked whether αKG supplementation could restore the activity of mTORC1 signaling in PHGDH-deficient TEMs. As expected, αKG supplementation largely increased the phosphorylation of S6 in PHGDH-deficient TEMs (Fig. 6G). Interestingly, treatment of BMDMs with αKG alone enhanced mTORC1 signaling activity (Fig. 6G). Taken together, these data show that the PHGDH-mediated de novoserine synthesis pathway modulates TAM polarization and proliferation at least in part *via* αKG and mTORC1 signaling.

### PHGDH depletion in macrophages reduces their crosstalk with tumor cells

**Fig. 7:**
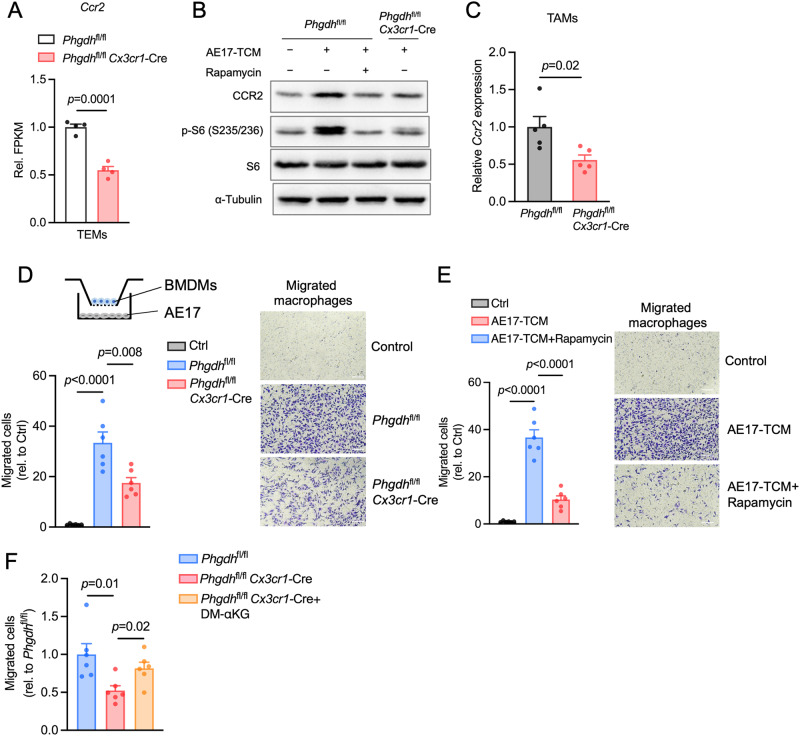
PHGDH depletion downregulates the expression of key chemotactic receptors on TAMs and impairs macrophage migration. **A** Relative FPKM (Rel. FPKM) of *Ccr2* in PHGDH-deficient TEMs and control TEMs (*n* = 4 biologically independent samples) according to RNA-seq. **B** Western blot analysis of the indicated proteins in PHGDH-deficient BMDMs and control BMDMs incubated with AE17-TCM supplemented with or without 100 nM rapamycin for 24 h. **C** qPCR analysis of *Ccr2* in TAMs sorted from mice with AE17 tumors (*n* = 5 tumors per condition). The data are expressed as the fold change relative to the WT. The results represent two independent experiments. **D** Scheme of the Transwell system with PHGDH-deficient BMDMs in the upper chamber and AE17 cells in the bottom chamber. The migration of macrophages was quantified 24 h after seeding (*n* = 6 random fields for each condition for two independent experiments with similar results). Scale bars, 100 µm. **E** Quantification of PHGDH-deficient macrophages incubated with or without 100 nM rapamycin that had migrated across a Transwell insert following coculture with AE17 cells (*n* = 6 random fields for each condition for two independent experiments with similar results). Scale bars, 100 µm. **F** Quantification of PHGDH-deficient macrophages that migrated across a Transwell insert following coculture with AE17 cells with or without 1 mM DM-αKG supplementation (*n* = 6 random fields for each condition for two independent experiments with similar results). Scale bars, 100 µm. The data are shown as the mean ± SEM (**A**, **D**–**F**). The data were analyzed using a two-tailed unpaired Student’s t test (A and C-F)

TAM recruitment and differentiation are key parts of tumor growth and malignancy. The attenuation of tumor growth and simultaneous reduction in TAM infiltration prompted us to assess the influence of PHGDH depletion in macrophages on the crosstalk of TAMs with tumor cells. Our RNA-seq data revealed that PHGDH depletion significantly downregulated the expression of *Ccr2*, a major chemoattractant receptor (Fig. 7A), while the expression of another chemoattractant receptor, CXCR4, was unaffected [32] (Supplementary Fig. S7A). Consistent with these findings, we confirmed that PHGDH deficiency markedly reduced CCR2 levels in TEMs treated with AE17-TCM or A549-TCM (Fig. 7B and Supplementary Fig. S7B) as well as in TAMs isolated from *Phgdh*^fl/fl^ *Cx3cr1*-Cre tumor-bearing mice (Fig. 7C). Given the important role of the CCL2-CCR2 chemokine signaling pathway in monocyte/macrophage recruitment in tumor models, we hypothesized that PHGDH depletion could impair macrophage migration toward cancer cells. Indeed, PHGDH-deficient macrophages exhibited defects in their ability to migrate toward AE17 and A549 cells (Fig. 7D and Supplementary Fig. S7C). Notably, rapamycin treatment not only downregulated CCR2 protein expression (Fig. 7B and Supplementary Fig. S7B) but also restricted the migration of TEMs toward cancer cells (Fig. 7E and Supplementary Fig. S7D), suggesting the potential involvement of mTORC1 signaling in CCR2-mediated macrophage migration. To gain further insight into the interplay between the PHGDH-mediated SSP and mTORC1 signaling in macrophage migration, we added αKG to the upper chambers of Transwell plates. Intriguingly, the additional αKG partially restored the migration ability of the PHGDH-deficient TEMs (Fig. 7F and Supplementary Fig. S7E). Thus, the interplay between PHGDH-mediated SSP and mTORC1 signaling may play a role in regulating macrophage migration to the TME.

To determine the levels of CCR2 expression in M1-like and M2-like TAMs in a more widespread manner, we mined the TCGA database using the TIMER2.0 online tool [33] (http://timer.cistrome.org). In many of the evaluated solid tumors (Spearman’s rank correlation coefficient, adjusted *p* value < 0.05), we observed a positive correlation between *Ccr2* gene expression and the infiltration of M1-like TAMs (Supplementary Fig. S7F, Supplementary Table S4). However, the correlation between *Ccr2* expression and the infiltration of M2-like TAMs was not as pronounced in most tumors (adjusted *p* value > 0.05) (Supplementary Table S4). Among the tumors with significant correlations (adjusted *p* value < 0.05), we found both positive and negative correlations between *Ccr2* expression and the infiltration of M2-like TAMs (Supplementary Fig. S7G, Supplementary Table S4). This is mainly because CCR2 is involved in the recruitment of monocytes/macrophages from the bloodstream to sites of inflammation or the TME. This recruitment process is a critical step in the generation of TAM populations within tumors, influencing the tumor immune landscape. Combined with our previous findings, one plausible explanation is that PHGDH deficiency diminishes CCR2 levels within TAMs, specifically affecting the monocyte/macrophage recruitment process without compromising the function of M1-like inflammatory TAMs. However, a rational understanding of the underlying mechanisms requires further exploration.

## Discussion

Increased serine uptake and de novo synthesis have emerged as critical regulators of cellular dynamics in cancer cells, lymphocytes, and endothelial cells [29, 34–38]. Dietary serine and glycine restriction agents or PHGDH inhibitors are being explored as promising antitumor therapies [39–43]. However, the role of PHGDH and serine metabolism in TAMs has not been determined. The protumorigenic impact of TAMs on the immunosuppressive TME and tumor progression suggests that targeting TAMs could be a valuable therapeutic approach [44]. In this study, we demonstrated that deletion of the serine biosynthesis enzyme PHGDH effectively suppressed the immunosuppressive activity of M2-like TAMs and two other different macrophage subsets, Mφ-c2-C1QA and Mφ-c3-APOE, in tumor tissues and attenuated tumor growth in a murine mesothelioma model. We found that both the typical Th2 immune response and the TME enhanced PHGDH-mediated de novo serine synthesis in macrophages and promoted immunosuppressive macrophage activation and proliferation through mTORC1 signaling. Depletion of PHGDH disrupted the enhanced metabolism critical for protumorigenic macrophage function, which resulted in a decrease in TAM infiltration and a phenotypic shift of M2-like TAMs toward an M1-like phenotype. Additionally, PHGDH deficiency reduced PD-L1 production in TAMs and partially restored antitumor T-cell immunity. Our observations revealed that the absence of PHGDH impaired M2-like TEM polarization, regardless of the PHGDH levels in the corresponding cancer cells. Nevertheless, we did not observe the inhibitory effects of PHGDH deficiency in older mice, which could be attributed to the overall decline in the immune system associated with aging. It is plausible that the immune system of older mice is less responsive to the PHGDH-dependent alterations that contribute to the observed antitumor effects in younger mice. These findings raise the intriguing possibility that targeting PHGDH in macrophages could serve as a promising therapeutic approach for solid tumors, particularly in younger individuals with low or negligible PHGDH expression in tumor cells.

Metabolism is increasingly recognized as a major regulator of macrophage function [45]. M1-like macrophages are characterized by a highly glycolytic metabolism, which enables extensive lactate secretion, NADPH and nucleotide biosynthesis, and reactive oxygen species (ROS) production, all of which support their cytocidal activities [46]. Conversely, M2-like macrophages predominantly rely on oxidative metabolism for bioenergetic purposes, which is associated with their role in tissue repair and remodeling [46]. Serine metabolism has recently been found to be involved in macrophage polarization in response to various environmental cues [16, 19, 20, 47, 48]. Yu et al. reported that LPS stimulation induces an increase in de novo serine synthesis in macrophages, which contributes to IL1β expression through the production of S-adenosylmethionine and histone methylation [20]. However, consistent with the findings of a recent investigation [48], our findings revealed that de novo serine synthesis did not change during the early time points (4 h and 8 h) after LPS stimulation, while the expression of two enzymes involved in one-carbon metabolism, namely, *Shmt1* and *Shmt2*, was markedly downregulated. We also found that prolonged stimulation (24 h) resulted in the downregulation of both *Phgdh* and *Psat*1 expression, indicating the dynamic effect of LPS on de novo serine synthesis. Interestingly, genetic ablation of *Phgdh* did not influence the expression of proinflammatory genes at 4 h or 8 h but led to increased expression levels at 24 h. Conversely, de novo serine synthesis was increased at 24 h following IL4 treatment or tumor CM stimulation, and genetic ablation of *Phgdh* significantly downregulated the expression of M2 markers. In agreement with these findings, recent data revealed that suppression of PHGDH enhances viral infection- or IFN-γ-activated M1 polarization but suppresses IL4-induced M2 polarization [49, 50]. Moreover, serine and glycine deprivation has been shown to downregulate the expression of the anti-inflammatory gene *Il10* and thus upregulate the expression of the proinflammatory genes *Il6* and *Tnfα* [47]. However, our study, along with others, revealed downregulated expression of the proinflammatory genes *Il1β* and *Tnfα* in the absence of exogenous serine and glycine, which may be attributed to the roles of serine in downstream glutathione (GSH) synthesis and the mTORC1 pathway [19, 48]. Our findings indicated that in contrast to M1 macrophage polarization, IL4-induced M2 polarization or TCM-induced protumorigenic macrophage polarization does not require exogenous serine. This was demonstrated by the lack of changes in the attenuated M2 phenotype when pharmacological or genetic inhibition of PHGDH was applied in medium supplemented with normal serine levels or in medium supplemented with additional serine or formate. These results suggest that factors other than serine or one-carbon production may play a crucial role in M2-like macrophage polarization. The addition of αKG successfully restored the expression of M2 markers in PHGDH- or PSAT1-deficient models, which highlights its role in regulating immunosuppressive macrophage polarization. Indeed, we found that both glutamate and αKG were markedly downregulated by PHGDH depletion in protumorigenic macrophages. Notably, conditional depletion of PHGDH resulted in a decrease in the serum level of αKG in tumor-bearing mice. Previous studies have shown that αKG is important for M2 macrophage activation through supporting JMJD3-dependent histone modification [18, 51]. In addition to these findings, our metabolomic and transcriptomic analyses revealed that serine biosynthesis-produced αKG is a determinant of mTORC1 signaling activation, which in turn supports immunosuppressive TAM activation, proliferation, and migration.

mTORC1 signaling has been found to activate serine and one-carbon metabolism through upregulating the expression of PHGDH, PSAT1 and SHMT [52]. Interestingly, recent studies have revealed that cellular serine levels also influence mTORC1 activation and downstream signaling in several cancer and immune cells [28, 29, 31, 48]. For example, in *Gclc*-mutant Tregs, elevated serine uptake and synthesis are required for cellular GSH compensation and mTORC1 activation, which in turn limits the immunosuppressive function of Tregs [29]. Serine deprivation has been shown to suppress macrophage IL-1β production through the inhibition of mTORC1 signaling in LPS-induced macrophages [48], which is different from what occurs in cancer cells, in which macrophages rely on enhanced de novo serine synthesis to promote mTORC1 signaling to support cell proliferation [28]. However, serine was abundantly available under our culture conditions and in vivo, suggesting that serine availability is not the limiting factor affecting mTORC1 signaling. Instead, we found that glutamine utilization in protumorigenic macrophages was markedly reduced by PHGDH ablation. Moreover, the suppression of PHGDH or PSAT1 significantly decreased the conversion of glutamate to αKG and decreased the cellular αKG pool in protumorigenic macrophages and in the serum of tumor-bearing mice. These findings suggest that αKG may play a regulatory role in mTORC1 signaling in TAMs. Indeed, we demonstrated that SSP-derived αKG activates mTORC1 signaling and contributes to the maintenance of an M2-like macrophage phenotype in the TME. Depletion of PHGDH affects αKG production and leads to suppression of mTORC1 signaling. Importantly, inhibition of mTORC1 signaling alone exhibited effects similar to those of genetic or pharmacological blockade of PHGDH, while supplementation with αKG rescued the defects associated with PHGDH deficiency and restored the recruitment of macrophages to cancer cells. In addition, we observed a reciprocal relationship between mTORC1 signaling and PHGDH, as evidenced by the downregulation of *Phgdh* expression in protumorigenic macrophages following rapamycin treatment. This finding suggested the presence of a positive feedback loop between PHGDH and the mTORC1 pathway. Preclinical studies targeting PHGDH have shown promising results in inhibiting the growth of cancers via amplification of PHGDH [34, 40–43]. It would be meaningful to investigate whether the inhibition of PHGDH alone or in combination with an mTORC1 inhibitor can elicit a promising antitumor response in patients whose tumor cells lack or express very low levels of PHGDH.

In conclusion, our study provides novel insights into the role of PHGDH-mediated serine biosynthesis in shaping the behavior of TAMs and their crosstalk with tumor cells. Depletion of PHGDH in macrophages led to reduced tumor growth, altered TAM polarization toward an antitumor phenotype, and improved antitumor T-cell immunity. Targeting PHGDH or modulating its downstream signaling pathways may be a novel strategy to manipulate TAM function and improve the efficacy of clinical immunotherapies.

### Limitations of the study

While early studies have established the pivotal role of *Cx3cr1* in macrophage functions, our experiments with older mice expressing *Phgdh*^fl/fl^ *Cx3cr1*-Cre did not reveal any signs of tumor alterations compared to those in *Phgdh*^fl/fl^ mice. Nevertheless, we acknowledge the possibility that age could influence how *Cx3cr1* impacts macrophage functions in our experiments. Overall, age has a strong effect on antitumor effects, and we consider this avenue highly interesting and worthy of thorough investigation in future studies.

## Methods

### Animals and tumor experiments

*Phgdh*^fl/fl^ mice were crossed with *Cx3cr1*^cre/+^ mice to obtain *Phgdh*^fl/fl^ *Cx3cr1*^cre/+^ (denoted as *Phgdh*^fl/fl^ *Cx3cr1*-Cre) or *Phgdh*^fl/fl^ *Cx3cr1*^+/+^ (denoted as *Phgdh*^fl/fl^) littermates. All mice in the study were bred on a C57BL/6 background and housed in conventional cages with access to a standard diet and water ad libitum at a constant ambient temperature and a 12-hour light cycle. For the tumor experiments, first, AE17 cells were injected intraperitoneally (i.p.) in mice to promote tumor growth. These recultured and STR-checked AE17 cells were subcutaneously injected (1 ×10^6^ in 100 μL of serum-free RPMI plus 25% Matrigel) into the right flank of 8- to 12-week-old *Phgdh*^fl/fl^ and *Phgdh*^fl/fl^ *Cx3cr1*-Cre mice. Tumor size was monitored at regular intervals of 2–3 days by measuring the length and width with a caliper. The tumor volume was calculated using the following formula: tumor volume = (length × width^2^)/2. When the tumors reached a size of 500–800 mm^3^, the animals were sacrificed by cervical dislocation, and the tumors were collected for further analysis. The experiments were repeated three times, with at least three mice per condition injected with tumor cells in each experiment. The therapeutic experiments conducted in this study were approved by the official Austrian ethics committee for animal experiments (ethics approval number: GZ 66.009/0134-V/3b/2019). Mice in individual experiments were sex- and age-matched.

### Cell culture

The human lung cancer cell Line A549, the human breast cancer cell line MDA-MB-231, the human colon cancer cell line HCT116, mouse RAW 264.7 macrophages, and the mouse fibroblast Line L929 were originally obtained from the ATCC. The mouse mesothelioma cell line AE17 was originally obtained from Sigma. All cancer cell lines and L929 cells were grown in Dulbecco′s modified Eagle′s medium (DMEM) high glucose mixture supplemented with 10% heat-inactivated bovine serum (FBS), 2 mM L-glutamine, 100 U/mL penicillin and 0.1 mg/mL streptomycin (complete DMEM). For metabolomic analysis, dialyzed heat-inactivated FBS was used. All the cell lines used in the study were confirmed to be free of mycoplasma contamination.

### Conditioned media preparation

A total of 0.25 ×10^6^ cancer cells were seeded in 15 mL of complete DMEM. After 24 h, the tumor-conditioned medium (TCM) was collected and centrifuged at 400 × *g* for 4 min. The supernatant was filtered through a 0.22-µm filter to eliminate debris and stored at −80 °C before use. For the L929-conditioned medium, a total of 17 × 10^6^ L929 cells were cultured in 40 mL of complete DMEM in 175 cm^2^ flasks. After 7 days, the L929 conditioned medium was collected, filtered (0.22 µm), aliquoted and stored at −20 °C until use.

### Macrophage isolation and in vitro polarization

BMDMs were generated as previously described [53]. Briefly, bone marrow cells were obtained by flushing the femurs and tibias of wild-type (WT), *Phgdh*^fl/fl^ or *Phgdh*^fl/fl^ *Cx3cr1*-Cre mice aged 6 to 8 weeks. After erythrocyte lysis with ammonium chloride-potassium (ACK) lysis buffer (0.15 M NH4Cl, 10 mM KHCO3 and 0.1 mM EDTA) for 5 min at room temperature (RT), the cells were washed twice with ice-cold phosphate-buffered saline (PBS), and the bone marrow cell suspension was cultured in BMDM differentiation medium (high glucose DMEM, 10% heat-inactivated FBS, 2 mM L-glutamine, 100 U/ml penicillin and 0.1 mg/ml streptomycin supplemented with 20% L929-conditioned supernatant) for 7 days. Fresh medium was added every 2–3 days. On Day 7, differentiated BMDMs (96% of the cells were positive for F4/80 and CD11b) were harvested and seeded in complete DMEM for different experiments.

To induce in vitro polarization, BMDMs were plated at a density of 1 ×10^6^ cells/well in six-well plates with 2 mL of DMEM and allowed to adhere overnight. The media was then replaced with TCM supplemented with 25 ng/mL IL-4 or 100 ng/mL LPS. After 4, 8 or 24 h of stimulation, the macrophages were collected for RNA or protein extraction. For tumor coculture experiments, AE17 cells were plated at a density of 3 × 10^5^ cells in a 24-well plate and allowed to attach overnight. Then, 4 × 10^5^ macrophages were seeded onto the cell inserts (0.3 μm pore size) for 10 min before they were added to the tumor cells. After stimulation with 100 ng/mL LPS for 24 h, the macrophages were collected for RNA extraction. For several experiments, macrophages were cultured with IL-4 in the presence or absence of 25 μM WQ-2101 (SML1970; Sigma), 0.4 mM serine (Sigma), 1 mM formate (Sigma) or 1 mM DM-αKG (Santa Cruz; sc-211344) for 24 h, after which the cells were harvested for further experiments as indicated.

### Flow cytometry of cells from tumor tissue

Tumors were excised, minced and dissociated in digestion buffer (1 mg/mL collagenase VIII and 1 mg/mL DNase I in PBS). The tissue was incubated for 30 min at 37 °C with agitation and then filtered through a 70 μm Falcon cell strainer to remove undigested tumor tissues. Red blood cells were lysed with ACK lysis buffer. Viable cells were counted after resuspension in WB buffer (5% FCS, 5 mM EDTA, and 20 μg/mL DNase I in PBS without Ca^2+^/Mg^2+^). Subsequently, 2–4 ×10^6^ cells in 100 μL of WB buffer were blocked with Fc blocking solution (anti-mouse CD16/CD32, 1:1000) for 10 min on ice. The cell suspension was then divided into two parts for macrophage staining and T-cell staining. For macrophage staining, the cell suspension was incubated in WB buffer for 30 min on ice with antibodies specific for CD45 (1:100, 30-F11; BioLegend), F4/80 (1:100, BM8; BioLegend), CD11b (1:100, M1/70; BioLegend), CD206 (1:100, C068C2; BioLegend), I-A/I-E (1:100, M5/114.15.2; BioLegend) and PD-L1 (1:100, 10 F.9G2; BioLegend). For T-cell staining, the cell suspension was incubated in WB buffer for 30 min on ice with antibodies specific for CD45 (1:100, 30-F11; BioLegend), CD3 (1:100, 17A2; BioLegend), CD25 (1:100, PC61; BioLegend), CD4 (1:100, GK1.5; BioLegend), and CD8a (1:100, QA17A07; BioLegend). For dead cell staining, Zombie Aqua (BioLegend) was added to the T cells before the incubation of antibodies, and 7-AAD (BioLegend) was added to the macrophages after the incubation of antibodies. For intracellular protein staining, Perm Fix solution was used for fixation and permeabilization, followed by FOXP3 (1:100, MF-14, BioLegend) and IFN-γ (1:100, XMG1.2, BioLegend) staining for 30 min at RT. To improve IFN-γ staining, exocytosis was inhibited by keeping the cells in the presence of brefeldin A (10 μg/mL, BioLegend) during the isolation and staining procedure. The data were obtained on a BD LSRFortessaTM X-20 cell analyzer (Becton Dickinson) and processed using FlowJo v10.

### TAM isolation using FACS

Tumors were isolated and digested as described above. The cell suspension was blocked with Fc blocking solution for 20 min on ice. The cells were subsequently incubated with fluorophore-conjugated antibodies against CD45, CD11b and F4/80 on ice in the dark for 30 min. After the incubation, the cells were washed with WB buffer and centrifuged at 300 *×g* for 5 min. The cell pellet was resuspended in 500 µL of WB buffer containing 7-AAD and sorted using a BD FACSMelody™ Cell Sorter. The CD45^+^CD11b^+^F4/80^+^ cells were sorted directly into TRI reagent (Thermo Fisher Scientific; 15596026). Then, RNA was isolated from the sorted cells following the manufacturer’s instructions.

### Cell cycle analysis

After treatment, the BMDMs were incubated with 20 μM BrdU (B5002, Sigma) in fresh culture medium at 37 °C in a humidified atmosphere (5% CO_2_). After 2 h, the cells were washed twice with PBS (without Ca^2+^/Mg^2+^) and harvested. Subsequently, 1 × 10^6^ cells were resuspended in cold (–20 °C) ethanol and fixed overnight at 4 °C. Afterward, the cells were washed and incubated in 2 M HCL containing 0.5% Triton X-100 at RT. After 30 min, the cells were washed again and incubated with 0.1 M Na2B4O7 for 10 min at RT. After another wash, the cell pellets were resuspended in anti-BrdU antibody solution (1:25, 3D4; BioLegend) and incubated for 30 min in the dark at RT. The cells were washed twice in cell staining buffer (BioLegend; 420201) and resuspended in 100 μL staining buffer containing 5 μL 7-AAD solution (BioLegend; 420403). After a 30 min incubation at RT in the dark, the cells were analyzed using a FACSAria II instrument (BD Biosciences).

### Immunohistochemistry and immunofluorescence of tumor sections

Tumor tissues were fixed in 4% formaldehyde, dehydrated, embedded in paraffin, and sectioned into 4 μm thick slices. To quench endogenous peroxidases, the sections were incubated with H_2_O_2_ (0.3% v/v) in PBS for 10 min. Antigen retrieval was performed by boiling the sections in 10 mM citrate buffer (pH 6.0) for 30 min. Prior to antibody incubation, the sections were blocked with Ultra V Block solution (UltraVision LP, Thermo Fisher Scientific) for 1 hour at RT. The sections were then incubated with a Ki67 antibody (1:200, 28074-1-AP, Proteintech) in a humid chamber for 1 hour at RT. Antibody binding was detected using the UltraVision LP detection system according to the manufacturer’s instructions. Subsequently, the sections were developed with DAB reagent (Dako) and counterstained with hematoxylin for 30 s. In addition, H&E staining was performed as previously described [54]. The stained slides were scanned using a whole slide scanner (Pannoramic SCAN II, 3DHISTECH). Evaluation and quantification of Ki67-stained cells were performed using Definiens software.

For immunofluorescence (IF) staining, tumor tissues were embedded and frozen in OCT medium (Sakura Finetek) and sliced into 4 µm sections using a cryomicrotome (Cryostar NX70, Thermo Fisher Scientific). The cryo-slides were then defrosted and fixed with 4% PFA. To block nonspecific binding, the tissue slides were incubated with a blocking solution (5% goat serum, 1% bovine serum albumin and 0.1% or 0.3% Triton X-100 in PBS). Subsequently, the slides were incubated with antibodies against CD68 (1:800, D4B9C, CST) and p-S6 (1:300, 50. Ser 235/236, Santa Cruz) overnight or with PHGDH (1:400, D3D5E, CST) and CD3-FITC (1:400, 17A2, CST) for 1.5 h. The samples were washed three times with PBS and subsequently incubated with fluorescence-labeled secondary antibodies for 1 hour in the dark. After incubation, the slides were counterstained with 4′,6-diamidine-2′-phenylindole dihydrochloride (DAPI; Sigma) for 10 min. The tissue slides were then mounted in nonhardening mounting medium (Vectashield Mounting Media, Vector). A confocal laser-scanning microscope (LSM 700, Carl Zeiss) equipped with a 63 Å/1.4 oil differential interference contrast M27 objective lens (Plan Apochromat, Carl Zeiss) was used to analyze the IF staining. The acquired images were processed using Zeiss ZEN 2.5 software.

### RNA extraction and RT‒qPCR

After treatment, BMDMs or TAMs were collected, washed twice with PBS and suspended directly in TRI reagent (Thermo Fisher Scientific). Total RNA was isolated following the manufacturer’s instructions. The concentration of RNA was determined using a Qubit 4 Fluorometer (Thermo Fisher Scientific), a Qubit RNA Broad Range Assay Kit (Invitrogen; Q10210), and a Nanodrop (Thermo Fisher Scientific). Reverse transcription of RNA was performed using the GoScript^TM^ Reverse Transcription Kit (Promega) with 1 μg of total RNA. The mRNA levels of the target genes were assessed using Luna Universal qPCR Master Mix (New England Biolab; M3003E) on a Bio-Rad CFX96 real-time system. The quantification of mRNA expression was performed via the comparative Ct (2^-∆∆Ct^) method. The Rps9 gene was used as the internal control. The primer pair sequences used are listed in Supplementary Table S2.

### Enzyme-linked immunosorbent assay (ELISA)

Differentiated BMDMs were seeded at a density of 1 ×10^6^ cells per well in a 6-well plate overnight. The media was then replaced with AE17-TCM, A549-TCM, MDA-MB-231-TCM or HCT116-TCM. After 24 h, cell-free supernatants were collected. The levels of secreted TGF-β and VEGF in the supernatants were determined following the manufacturer’s instructions using a TGF-β ELISA kit (Invitrogen, BMS608-4) and a VEGF ELISA kit (Sigma, RAB0509-1KT). Respectively. The experiment was performed in three biological replicates.

### Cell viability assay

*Phgdh*^fl/fl^ *Cx3cr1*-Cre and *Phgdh*^fl/fl^ BMDMs were plated at a density of 1 ×10^4^ cells/well into a 96-well plate for 24 h. The media were then replaced with 100 μL of fresh DMEM containing 10% CCK8 (MedChemExpress, HY-K0301) for 1 hour. The absorbance was measured at 450 nm by a microplate reader (Thermo Scientific).

### Proliferation assay (CCK8)

*Phgdh*^fl/fl^ *Cx3cr1*-Cre, *Phgdh*^fl/fl^ or WT BMDMs were seeded at a density of 5 ×10^3^ cells/well into a 96-well plate and incubated overnight. The media were subsequently replaced with AE17-TCM, A549-TCM, or AE17-TCM supplemented with 100 nM rapamycin (MedChemExpress, HY-10219). CCK8 solution was added as described above, and the absorbance at 450 nm was measured every 24 h for a period of 3 days.

### Western blot analysis

Macrophages were homogenized in cold RIPA lysis buffer containing protease inhibitor cocktails (Sigma; 4693116001) and phosphatase PhosStop EASYPack cocktails (Roche; 4906837001) followed by sonication with a tip-probe sonicator on ice. The concentrations of the proteins were determined using the bicinchoninic acid (BCA) assay. Equal amounts of proteins were loaded onto 10% or 12% SDS‒PAGE gels, separated by electrophoresis, and then transferred onto polyvinylidene fluoride (PVDF) membranes. The membranes were subsequently incubated with primary antibodies. The protein bands were visualized using an enhanced chemiluminescence (ECL) system (Amersham Biosciences, Cytiva) and imaged using an iBright FL1500 Imaging System (Thermo Fisher). The primary antibodies used were anti-ARG1 (1:1000, sc-47715), p-ribosomal protein S6 (1:1000, sc-293144), anti-ribosomal protein S6 (1:1000, sc-74459), and anti-p-4E-BP1 (1:1000, sc-293124); anti-4E-BP1 (1:1000, sc-81149); anti-p-AKT1/2/3 (1:1000, sc-81433); and anti-AKT1/2/3 (1:1000, sc-81434) obtained from Santa Cruz; and anti-PHGDH (1:750, 13428), anti-CCR2 (1:1000, 12199) obtained from CST; and anti-α-Tubulin (1:1000, 1224-1-AP) and secondary antibodies horseradish peroxidase (HRP)-conjugated anti-rabbit Ig (H + L) (1:5000, SA00001-2) and HRP-conjugated anti-mouse Ig(H + L) (1:5000, SA00001-1) were purchased from Proteintech.

### Macrophage migration assay

In vitro migration assays were performed using tissue culture-treated cell inserts with 8.0 µM pore size PET membranes (cellQART). A549 or AE17 cancer cells were seeded at a density of 3 ×10^5^ cells/well in a 24-well plate and incubated overnight. For some experiments, 100 nM rapamycin or 1 mM DM-αKG was added to the upper chamber. The media were then removed, and the cells were washed twice with PBS. Fresh medium containing 0.5% heat-inactivated FBS was added to the wells. Next, 0.5 × 10^6^ *Phgdh*^fl/fl^ *Cx3cr1*-Cre or *Phgdh*^fl/fl^ BMDMs were seeded in 200 μL DMEM supplemented with 0.5% heat-inactivated FBS on the top of the membrane chamber and allowed to incubate for 10 min before the chamber was submerged into the lower compartment of the 24-well plate. After 24 h, the insert was removed, washed and fixed in 4% paraformaldehyde (PFA) for 20 min. Nonmigrated cells on the upper side of the membrane were removed with cotton swabs, and the insert was then submerged in 0.2% crystal violet solution for 10 min for cell staining. The insert was dried overnight following washing with tap water. The migrated cells were photographed in 6 random fields and quantified manually by counting single cells under a Nikon ECLIPSE Ti2 microscope (Nikon).

### Seahorse extracellular flux analysis

The macrophage OCR and ECAR were measured as previously described [55]. Briefly, *Phgdh*^fl/fl^ *Cx3cr1*-Cre and *Phgdh*^fl/fl^ BMDMs were plated in 6-well plates at a density of 1 × 10^6^ cells per well. After incubating with TCM for 24 h, the cells were scraped and transferred to Cell-Tak (Corning)-precoated XF24e-cell culture plates for immediate adhesion with XF assay medium (Agilent Technologies). Before the measurements were taken, the cells were placed in a CO_2_-free incubator at 37 °C for 1 hour. In the meantime, a utility plate containing 1 mL of calibrant solution was prepared to hydrate the XF extracellular flux cartridge. The utility plate was incubated overnight in a CO_2_-free incubator at 37 °C. Following the instructions of the glycolysis stress assay from Agilent, glucose (30 mM), oligomycin (30 μM) and 2-deoxy-D-glucose (2-DG, 50 mM) were added to each well. Real-time extracellular acidification (ECAR) was measured using a seahorse analyzer. For the mitochondrial stress assay, the cells were incubated in XF assay medium supplemented with 2 mM glutamine, 1 mM pyruvate and 25 mM glucose. The final concentrations of oligomycin (3 μM), FCCP (3 μM), and a mixture of rotenone/antimycin A (0.5 μM) were injected into ports A, B, and C, respectively. The data were monitored for the ECAR and OCR, and the values were normalized to the cell number.

### RNA-seq and bioinformatics analysis

*Phgdh*^fl/fl^ *Cx3cr1*-Cre and *Phgdh*^fl/fl^ BMDMs were seeded in a 6-well plate at a density of 1 ×10^6^ cells per well. After incubating with AE17-TCM for 24 h, total RNA was isolated using TRIzol reagent according to the manufacturer’s instructions. Library preparation was carried out at the Next Generation Sequencing (NGS) unit of the Vienna Biocenter Core Facility (VBCF, Vienna, Austria). The NEBNext Ultra Directional RNA Library Prep Kit (New England Biolabs) was used for preparing the sequencing libraries. The average length of the pooled libraries was 330–360 bp, and the libraries were sequenced on a NextSeq550 instrument (Illumina) using the 1 ×75 bp sequencing mode. The RNA-Seq reads were mapped to the reference mouse mm10 genome using TopHat2 [56]^,^ and fragments per kilobase of transcript per million fragments mapped (FPKM) were calculated via HT-seq [57]. The differential gene expression analysis was conducted by DESeq2 [58] with the following statistical cutoffs: genes with low counts greater than 10, adjusted *p* value less than 0.05, and an absolute log2-fold change greater than 1.5.

### siRNA transfection

*Psat1* siRNA was obtained from Santa Cruz, and siRNA transfection was performed as described earlier [55]. Briefly, RAW 264.7 macrophages were seeded onto a 6-well plate at a density of 0.6 × 10^6^ cells/well. Once the cells reached 70–80% confluence, they were transfected with either control siRNA or *Psat1* siRNA using Lipofectamine 3000 Transfection Reagent (Invitrogen) according to the manufacturer’s instructions. After 48 h of transfection, the cells were gently washed with PBS and then incubated in AE17-TCM supplemented with or without DM-αKG for 24 h. The cells were subsequently harvested for analysis of mRNA expression and metabolites.

### Pulsed stable isotope-resolved metabolomics

Stable isotope tracing experiments were performed as described previously with some modifications [59]. Briefly, differentiated BMDMs were seeded at a density of 1 ×10^6^ cells per well in DMEM and incubated overnight. The medium was then replaced with AE17-TCM, and the cells were incubated for 24 h. Two hours prior to harvest, a second medium was used to expose the cells to DMEM without glucose or serine (D9802-01, USBiological) supplemented with 4.5 g/L U-[^13^C]-glucose (Cambridge Isotope Laboratories; CLM-1396), 0.4 mM U-[^13^C]-serine (Cambridge Isotope Laboratories; CLM-1574-H) or 1 mM U-[^13^C]-glutamine (Cambridge Isotope Laboratories; CLM-1822-H). The cells were washed twice with HEPES buffer (5 mM HEPES, 100 mM NaCl, pH 7.4) and quenched by adding 1 mL of 50% precooled methanol (−80 °C) containing 2.5 nM phenyl β-D-glucopyranoside (Sigma) as an internal control. Cell lysates were collected in polypropylene tubes by scraping, followed by the addition of 200 μL of chloroform. The samples were shaken for 1 hour at 4 °C. After centrifugation, the supernatant was transferred to a new tube and dried in a SpeedVac (Labogene). Next, 15 μL of methoxyamine hydrochloride solution (40 mg/mL in pyridine) was added to the dried fraction, and the mixture was then incubated for 90 min at 30 °C. Subsequently, 60 μL of N-methyl-N-trimethylsilyltrifluoroacetamide (MSTFA) was added, and the mixture was incubated for 30 min at 37 °C. The reaction mixture was centrifuged for 10 min at 4 °C and 21,000 × *g*, after which the supernatants were transferred to glass vials with microinserts. The metabolites were measured using GC‒MS following standard protocols [60]. Data processing and natural ^13^C labeling correction were performed using the Data Extraction for Stable Isotope-labeled metabolites (DExSI) software [61]. The default settings were used except for the following parameters. The number of points on either side of the apex and scan window was set to 10. Mass isotopomer fraction labeling was determined by integrating metabolite ion fragments (Supplementary Table S3).

### Metabolomic analysis by GC‒MS and LC‒MS

Cellular metabolites were extracted and analyzed according to previously established methods with modifications [53]. Briefly, *Phgdh*^fl/fl^ *Cx3cr1*-Cre and *Phgdh*^fl/fl^ BMDMs were seeded at a density of 1 × 10^6^ cells/well in DMEM and incubated overnight. The medium was then replaced with AE17-TCM for 24 h. The cells were washed twice with 0.9% NaCl and quenched by adding 80% ice-cold methanol containing 2.5 nM phenyl β-D-glucopyranoside (GC‒MS) or 10 nM ampicillin (LC‒MS) as the internal extraction standard. The extraction samples were incubated for 15 min at 4 °C and then centrifuged for 10 min at 21,000 × *g*. The supernatant was transferred to a fresh polypropylene tube and dried in a SpeedVac. The cell pellets were lysed in RIPA buffer, after which the protein levels were measured for normalization purposes. For measurements of αKG, serine and glycine levels in the blood of *Phgdh*^fl/fl^ *Cx3cr1*-Cre and *Phgdh*^fl/fl^ tumor-bearing mice, blood was drawn weekly from the facial vein and incubated for 2 h at 4 °C. Then, 10 μL of serum was collected after centrifugation, and metabolites were extracted in 1 mL of precooled methanol. The extraction samples were incubated for 15 min at 4 °C, and then centrifuged for 10 min at 21,000 × *g*. The supernatants were subsequently dried in a SpeedVac system. For GC‒MS analysis, sample derivatization was carried out as described above. The injection volume of each sample was 1 μL, and the samples were injected at a 1:5 split ratio. The total ion chromatogram was deconvoluted, and peak alignment and integration were performed using the software MS-DIAL [62].

LC‒MS analysis was performed using an UltiMate 3000 UHPLC System (Thermo Scientific) coupled to an LTQ-Orbitrap Elite mass spectrometer (Thermo Scientific). The extracted samples were dissolved in MS buffer (10 mM NH_4_OAc in mqH_2_O and 2% methanol, pH 6.9) and then centrifuged for 10 min at 21,000 × *g*. The supernatant was subsequently transferred to HPLC vials. The sample was separated using a Thermo Scientific^TM^ Accucore™ Vanquish C-18+ (100 × 2.1 mm, 1.5 µm particle size) UHPLC column equipped with a Thermo Scientific^TM^ Accucore™ Defender C18 guard cartridge (10 × 2.1 mm, 2.6 µm particle size). The sample injection volume was 5 µL. The mobile phase system consisted of a mixture of solvent A (10 mM NH4OAc in mqH2O, pH 6.9) and solvent B (LC‒MS grade methanol). A gradient elution method was used for the analysis: 0–1 min 5% B, 5–30 min linear gradient to 85% B, 0.1 min back to 0% B, 10 min 0% B. The flowrate was set to 0.25 mL/min, and the column oven temperature was 30 °C. MS analysis was performed with the following ion source parameters: spray voltage, 4 kV; capillary temperature, 350 °C; sheath gas, 35; and auxiliary gas, 10. Mass spectrometry was performed in positive ion mode. The full MS scan range was 100–1200 m/z with a resolution of 120,000. The normalized collision energy for collision-induced dissociation (CID) was set to 35%. Xcalibur software (Thermo Scientific) and MS-DIAL were used for data analysis and interpretation.

### Statistical analysis

All of the data are presented as mean ± SEM unless otherwise noted. All of the statistical analyses were performed using Prism v9 (GraphPad Software) or Excel (Microsoft). Statistical significance was analyzed using an unpaired Student’s *t* test or two-way ANOVA with Tukey’s multiple comparisons test. Differences with *p*  <  0.05 were considered to be statistically significant.

## Supplementary information

TableS1

TableS4

Supplemental material

Uncropped gels

## Data Availability

All of the data from this manuscript are available from the corresponding author upon request. RNA sequence data that support the findings of this study have been deposited in the Gene Expression Omnibus (GEO) under accession number GSE 236357.
