# Supplementary material for: Targeting PHGDH reverses the immunosuppressive phenotype of tumor-associated macrophages through α-ketoglutarate and mTORC1 signaling
Source: Cell Mol Immunol. 2024 Feb 27;21(5):448–65. doi: 10.1038/s41423-024-01134-0 (PMC11061172; doi:10.1038/s41423-024-01134-0)

Supplemental information

**Targeting PHGDH reverses the immunosuppressive function in tumor-associated macrophages through α-ketoglutarate and mTORC1 signaling**

Zhengnan Cai, Wan Li, Sonja Hager, Jayne Louise Wilson, Leila Afjehi-Sadat, Elke H. Heiss, Thomas Weichhart, Petra Heffeter, Wolfram Weckwerth^*^

**Supplemental table legends**

**Table S1. All identified and quantified metabolites obtained from GC-MS and LC-MS**

**Table S2. Primers used for RT-qPCR**

**Table S3. Metabolite specific mass fragments for the calculation of ^13^C-isotope incorporation**

**Table S4. Correlation between *Ccr2* gene expression and TAM infiltration in diverse cancer types**

**Supplemental figure legends**

**Figure S1. PHGDH supports M2 immunosuppressive phenotype**. (**A**) Gating strategies for TAMs cell sorting analyses. Flow cytometry plot axes are displayed in logarithmic scale, except for forward scatter (FSC) and side scatter (SSC) for which a linear scale was used. (**B**) Western blot analysis of ARG1 and PHGDH protein expressions in WT BMDMs treated with 20 ng/mL IL-4 or DMEM. (**C**) Cell viability analysis of BMDMs from PHGDH-deficient mice and *Phgdh*^fl/fl^ mice (*n* = 4 biologically independent samples per genotype). (**D-E**) qPCR analysis of *Psat1*, *Shmt1* and *Shmt2* in PHGDH-deficient BMDMs and *Phgdh*^fl/fl^ (control) BMDMs stimulated with 20 ng/mL IL-4 (D)or 100 ng/mL LPS (E) for 4 h, 8 h and 24 h (*n* = 3 independent experiments). Data are expressed as the fold change relative to control (DMEM treated WT macrophages) at indicated time points. (**F-J**) qPCR analysis of *Slc1a4* (F) and *Slc1a5* (J) in WT BMDMs stimulated with 100 ng/mL LPS, 20 ng/mL IL-4 or DMEM for 4 h and 24 h (*n* = 3 independent experiments). (**H**) qPCR analysis of *Il1b* in WT BMDMs stimulated with 100 ng/mL LPS or 100 ng/mL LPS pretreated with 50 μM L-phenylglycine (l-phg) (*n* = 3 independent experiments). (**I**) qPCR analysis of *Arg1* in WT BMDMs stimulated with 20 ng/mL IL-4 or 20 ng/mL IL-4 pretreated with 50 μM l-phg (*n* = 3 independent experiments). Data are shown as the mean ± SEM. Statistical significance was calculated using a two-tailed unpaired Student’s t-test.

**Figure S2. PHGDH deficiency diminishes M2-like TEMs phenotype**. (**A**) Western blot detection of PHGDH in AE17, A549, MDA-MB-231 and HCT116 cancer cells. (**B-C**) Western blot detection of ARG1 and PHGDH in *Phgdh*^fl/fl^ *Cx3cr1*-Cre BMDMs and *Phgdh*^fl/fl^ BMDMs incubated with DMEM, MDA-MB-231-TCM (B) or HCT116 (C) for 24 h. (**D-E**) Extracellular levels of serine (D) and glycine (E) in conditioned media from WT BMDMs incubated with DMEM, AE17-TCM, A549-TCM or MDA-MB-231-TCM for 24 h followed by GC-MS (*n* = 3 biologically independent samples). Data are expressed as the fold change relative to the unconditioned DMEM. (**F**) Images of AE17-TCM- or A549-TCM-stimulated PHGDH-deficient for 2 days. Scale bar, 100 µm. (**G**) Western blot detection of pro.caspase-3 and cl.caspase-3 in *Phgdh*^fl/fl^ *Cx3cr1*-Cre BMDMs and *Phgdh*^fl/fl^ BMDMs incubated with DMEM or AE17-TCM. *Phgdh*^fl/fl^ BMDMs was treated with 400 μM palmitic acid (PA.) for 24 h as the positive control. (**H**) PHGDH-deficient BMDMs and control BMDMs were treated with AE17-TCM or A549-TCM for the indicated days followed by 1h CCK8 incubation (*n* = 4 biologically independent samples). The optical density (OD) was detected at 450 nm. Data are shown as the mean ± SEM. Statistical significance was calculated using a two-tailed unpaired Student’s t-test.

**Figure S3. PHGDH deficiency in macrophages favors antitumor immunity.** (**A**) Representative images of tumors from AE17 tumor-bearing mice. (**B**) Gating strategies for flow cytometry analyses described in Figures 3D, 3G and S3E. Flow cytometry plot axes are presented in logarithmic scale, except for forward side scatter (FSC) and side scatter (SSC) for which a linear scale was used. (**C**) qPCR analysis of selected macrophage markers in TAMs sorted from AE17 mesothelioma (*n* = 5 tumors per condition). Data are expressed as the fold change relative to *Phgdh*^fl/fl^ samples. Results represent two independent experiments. Data are shown as the mean ± SEM. Statistical significance was calculated using a two-tailed unpaired Student’s t-test.

**Figure S4.** (**A**) Analysis of the proportion of T cells (CD3^+^), CD4 (CD4^+^) and CD8 (CD8^+^) from AE17 mesothelioma (*n* = 10 tumors per condition). Results represent three independent experiments. (**B**) Representative images of cyrosectioned AE17 tumors stained with antibodies against PHGDH (red) and CD3 (green) as well as the nuclear stain DAPI. Scale bar, 20 μm. Data are shown as the mean ± SEM. Statistical significance was calculated using a two-tailed unpaired Student’s t-test.

**Figure S5. PHGDH deficiency restrains M2-like TEMs functions.** (**A-C**) GSEA plots of the E2F targets (A), p53 pathway (B) and inflammatory response (C) gene signature in *Phgdh*^fl/fl^ *Cx3cr1*-Cre TEMs relative to *Phgdh*^fl/fl^ TEMs from the analysis in Figure 4B with their respective normalized enrichment scores (NES) and false discovery rates (FDR). (**D**) KEGG enrichment analysis conducted on *Phgdh*^fl/fl^ *Cx3cr1*-Cre TEMs and *Phgdh*^fl/fl^ TEMs using GSEA. (**E**) Principal Component Analysis (PCA) of total identified metabolites in *Phgdh*^fl/fl^ TEMs and PHGDH-deficient TEMs. Samples are plotted based on the first two principal components (PC 1 and PC 2) with the indicated contributions to total variance (*n* = 4 biologically independent samples). (F) qPCR analysis of representative genes encoding for subunits of the five protein complexes involved in the ETC in TAMs sorted from AE17 mesothelioma (*n* = 5 tumors per condition). Data are expressed as the fold change relative to *Phgdh*^fl/fl^ samples. Results represent two independent experiments. (G) qPCR analysis of representative genes encoding for glycolytic pathway in TAMs sorted from AE17 mesothelioma (*n* = 5 tumors per condition). Data are expressed as the fold change relative to *Phgdh*^fl/fl^ samples. Results represent two independent experiments. (H) qPCR analysis of *Psat1* and *Psph* in TAMs sorted from AE17 mesothelioma (*n* = 5 tumors per condition). Data are expressed as the fold change relative to *Phgdh*^fl/fl^ samples. Results represent two independent experiments. Data are shown as the mean ± SEM. Statistical significance was calculated using a two-tailed unpaired Student’s t-test.

**Figure S6. mTORC1 signaling regulates M2-like TEM polarization and proliferation.** (**A**) Heatmap of gene signatures for mTORC1 signaling in Figure 6A. (**B**) Protein quantification of S6 (p-S235/236) shown in Figure 6B (*n* = 3 biologically independent samples). Data are presented as fold changes relative to the untreated group. Data are normalized on tubulin and the total S6 protein. (**C**) Western blot detection of indicated proteins in WT BMDMs incubated with A549-TCM supplying with 100 nM rapamycin or 25 μM WQ-2101 for 24 h. (**D**) Protein quantification of S6 (p-S235/236) shown in Figure S6C (*n* = 3 biologically independent samples). Data are presented as fold changes relative to the untreated group. Data are normalized on the tubulin and the total S6 protein. (**E**) *Phgdh*^fl/fl^ BMDMs was treated with A549-TCM for the indicated days followed by 1h CCK8 incubation (*n* = 4 biologically independent samples). The OD was detected at 450 nm. Data were normalized to the control (DMEM-treated *Phgdh*^fl/fl^ BMDMs) and expressed as fold change. Data are shown as the mean ± SD (C). Data analyzed using a two-way ANOVA with Tukey’s multiple comparisons test (C).

**Figure S7.** **PHGDH-mediated SSP interacts with mTORC1 signaling to regulate macrophage migration.** (**A**) Relative FPKM (Rel. FPKM) of *Cxcr4* in PHGDH-deficient TEMs and control TEMs (*n* = 4 biologically independent samples). (**B**) Western blot detection of indicated proteins in PHGDH-deficient BMDMs and control BMDMs incubated with A549-TCM supplying with or without 100 nM rapamycin for 24 h. (**C**) Scheme of the transwell system with PHGDH-deficient BMDMs in the upper chamber and A549 cells in the bottom. Migration of macrophages was quantified 24 h after seeding (*n* = 6 random fields for each condition for two independent experiments with similar results). Scale bars, 100 µm. (**D**) Quantification of PHGDH-deficient macrophages incubating with or without 100 nM rapamycin that have migrated across a transwell filter following co-culture with AE17 cells (*n* = 6 random fields for each condition for two independent experiments with similar results). Scale bars, 100 µm. (**E**) Quantification of PHGDH-deficient macrophages that have migrated across a transwell filter following co-culture with A549 cancer cells, with or without supplementation of 1 mM DM-αKG (*n* = 6 random fields for each condition for two independent experiments with similar results). (**F**) Spearman’s correlation between *Ccr2* gene expression and M1-like TAM infiltration based on CIBERSORT algorithm. (**G**) Spearman’s correlation between *Ccr2* gene expression and M2-like TAM infiltration based on CIBERSORT algorithm. Purity adjustment was applied when analyzing the TCGA database. Only significant correlations are displayed, with an adjusted *p*-value < 0.05. Data are shown as the mean ± SEM (A and C-D). Data analyzed using a two-tailed unpaired Student’s t-test (A and C-E).

**Table S2. Primers used for RT-qPCR**

| Target | Primer Forward | Primer Reverse |
| --- | --- | --- |
| *Arg1* | ACATTGGCTTGCGAGACGTA | ATCGGCCTTTTCTTCCTTCCC |
| *Mrc1* | CTCTGTTCAGCTATTGGACGC | CGGAATTTCTGGGATTCAGCTTC |
| *Ym1* | CCAGCAGAAGCTCTCCAGAAGCA | TGGTAGGAAGATCCCAGCTGTACG |
| *Il10* | GCTCTTACTGACTGGCATGAG | CGCAGCTCTAGGAGCATGTG |
| *Nos2* | CAGAGGACCCAGAGACAAGC | TGCTGAAACATTTCCTGTGC |
| *Il1b* | TGGCAACTGTTCCTG | GGAAGCAGCCCTTCATCTTT |
| *Tnfa* | TCTGTCTACTGAACTTCGGGGTGA | TTGTCTTTGAGATCCATGCCGTT |
| *Tgfb* | TGACGTCACTGGAGTTGTACGG | GGTTCATGTCATGGATGGTGC |
| *Past1* | AGAAGAATGTTGGCTCTGCC | CCCATGACGTAGATGCTGAA |
| *Phgdh* | CAGGTGGTTACACAAGGAACA | GTCTGCCTGCTTAGATGCTT |
| *Shmt1* | CCACGCTCCTAATACAAGGCA | TGCTGTAAACCTCGGCATCA |
| *Shmt2* | GCGGATGTTGTTACCACC | GGGAACACAGCGAAGTTGAT |
| *Rps9* | GCAAGATGAAGCTGGATTAC | GGGATGTTCACCACCTG |
| *Mgl1* | TGCAACAGCTGAGGAAGGACTTGA | AACCAATAGCAGCTGCCTTCATGC |
| *Mgl2* | GCATGAAGGCAGCTGCTATTGGTT | TAGGCCCATCCAGCTAAGCACATT |
| *Ccr2* | TTTGTTTTTGCAGATGATTCAA | TGCCATCATAAAGGAGCCAT |
| *Il6* | ACAAAGCCAGAGTCCTTCAGAGAG | TTGGATGGTCTTGGTCCTTAGCCA |
| *Thbs1* | GGGGAGATAACGGTGTGTTTG | AGACTCTGGAATGCGGTTG |
| *Slc40a1* | TGGAACTCTATGGAAACAGCCT | TGGCATTCTTATCCACCCAGT |
| *Gpnmb* | CATTCCCATCTCGAAGGTGAAA | AAATGGCAGAGTCGTTGAGGA |
| *Apoe* | GACCCAGCAAATACGCCTG | CATGTCTTCCACTATTGGCTCG |
| *Lst1* | TGGAAAGGAATGCCCAGGTC | GGCATAGTCAGTGCTGAGGT |
| *Fcgr3* | AATGCACACTCTGGAAGCCAA | CACTCTGCCTGTCTGCAAAAG |
| *Atp5a1* | GAGACTGGGCGTGTGTTAAG | CTCCTCTGCTTGAACATTCCTC |
| *Cox5a* | ATGCCTGGGAATTGCGTAAAG | TGCGAACAGCACTAGCAAAAT |
| *Gapdh* | TGGCCTTCCGTGTTCCTAC | GAGTTGCTGTTGAAGTCGCA |
| *Hk2* | TGATCGCCTGCTTATTCACGG | AACCGCCTAGAAATCTCCAGA |
| *Ldha* | TGTCTCCAGCAAAGACTACTGT | GACTGTACTTGACAATGTTGGGA |
| *Ndufb8* | TGTTGCCGGGGTCATATCCTA | AGCATCGGGTAGTCGCCATA |
| *Pfkm* | GCGACTTGCTGAATGATCTCC | CATTGTCGATTGAGCCAACCA |
| *Pgam1* | GTCCTGGATGCCATTGACCA | GACCTGTCAGACCGCCATAG |
| *Sdhb* | AATTTGCCATTTACCGATGGGA | AGCATCCAACACCATAGGTCC |
| *Pkm* | GTGGGCAGCAAGATCTACGT | GAGCCACCATTCTCCACCTC |
| *Psph* | GGATGCAGTGTGCTTTGATGT | CCAGCTCCCTTATGCCAGG |
| *Uqcrc2* | AAAGTTGCCCCGAAGGTTAAA | GAGCATAGTTTTCCAGAGAAGCA |

**Table S3. Metabolite specific mass fragments for the calculation of ^13^C-isotope incorporation**

| Compound | Derivate | Ions | Formula | Maximum labellled atoms | Labelling with |
| --- | --- | --- | --- | --- | --- |
| Serine | 3TMS | 218, 219, 220 | C8H20N1O2Si2 | C2 | U-[^13^C]-glucose or serine |
| αKG | 1MeOX 2TMS | 304, 305, 306, 307, 308, 309 | C11H22O5N1Si2 | C5 | U-[^13^C]-glutamine |

Figure S1


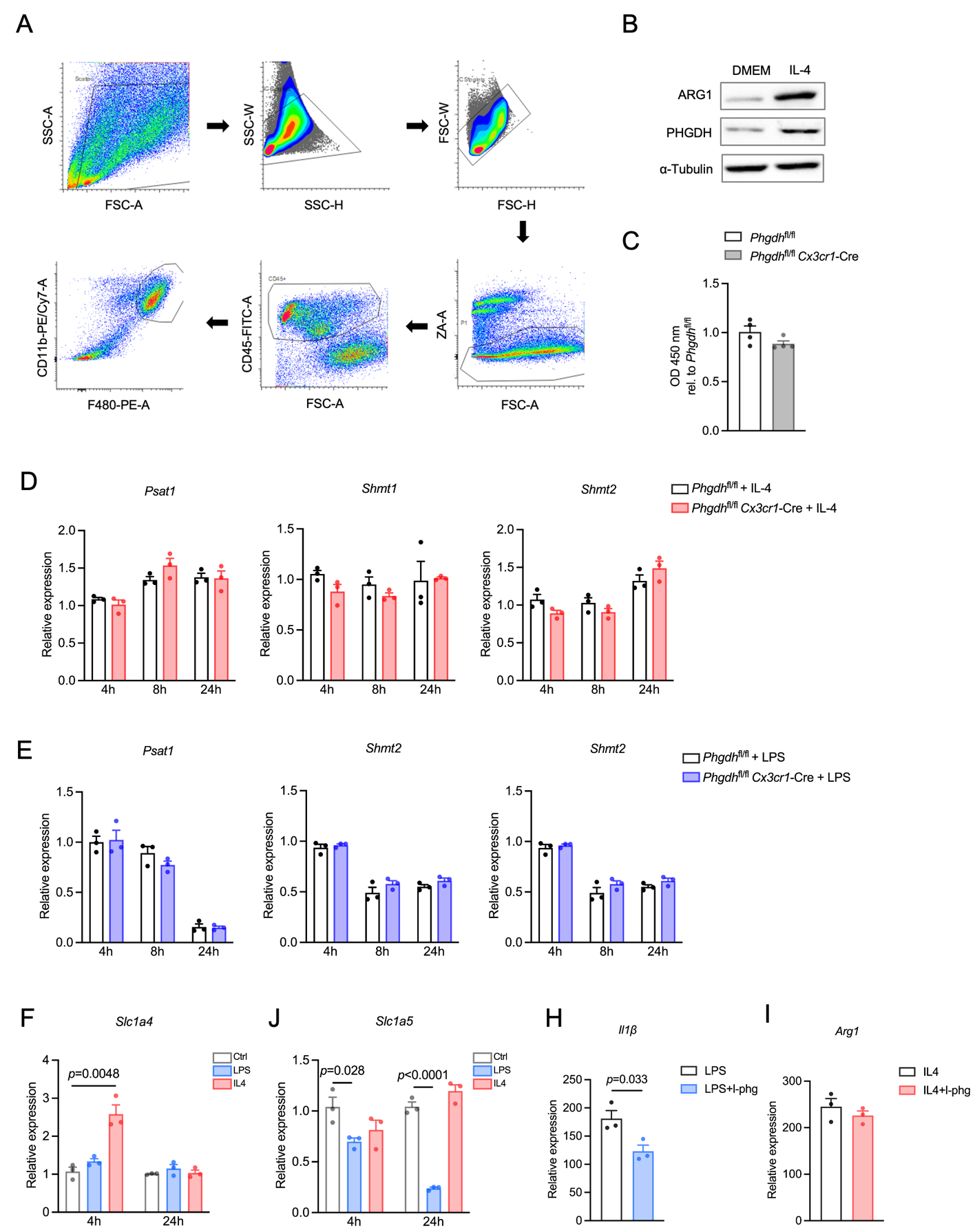


Figure S2


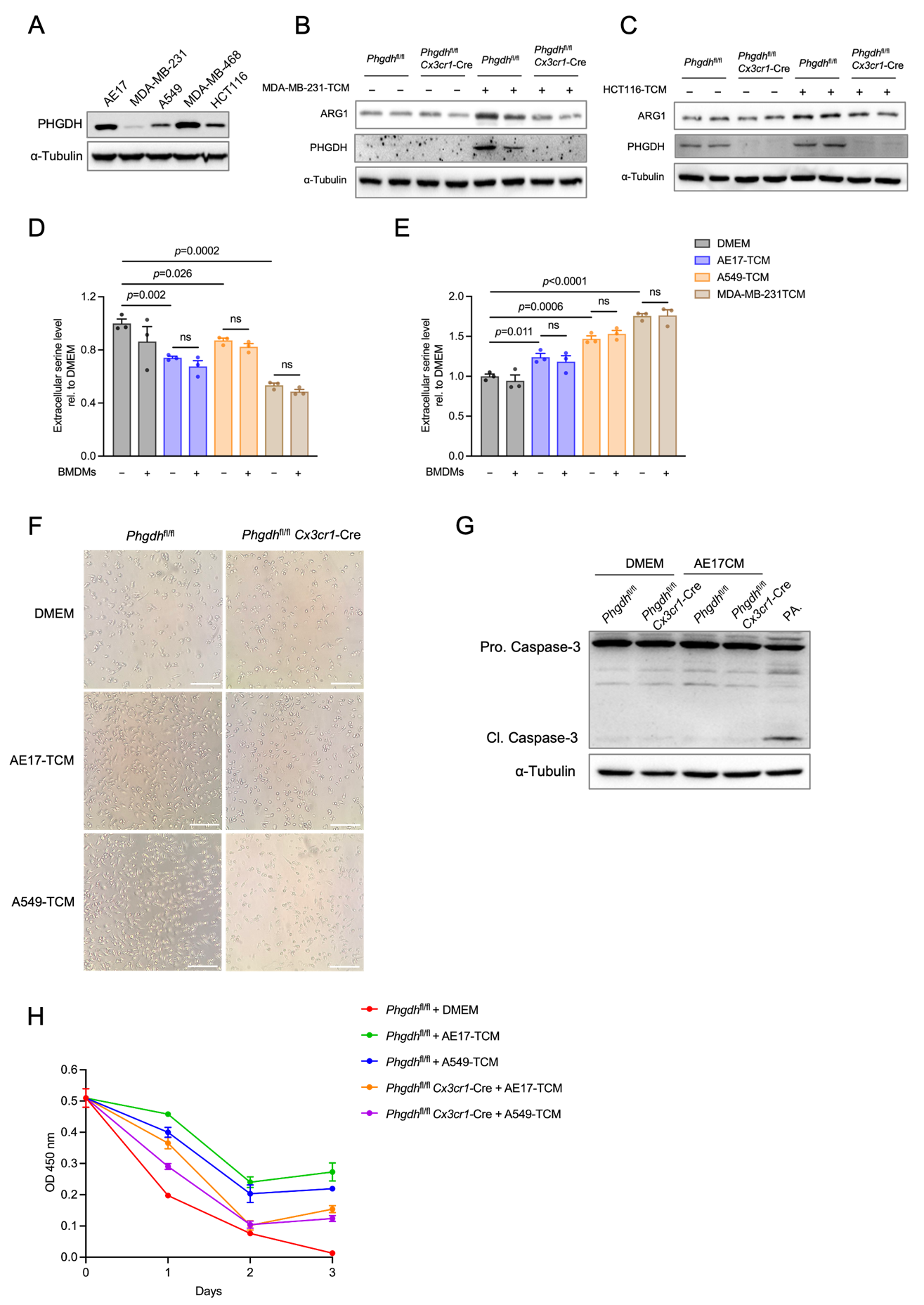


Figure S3


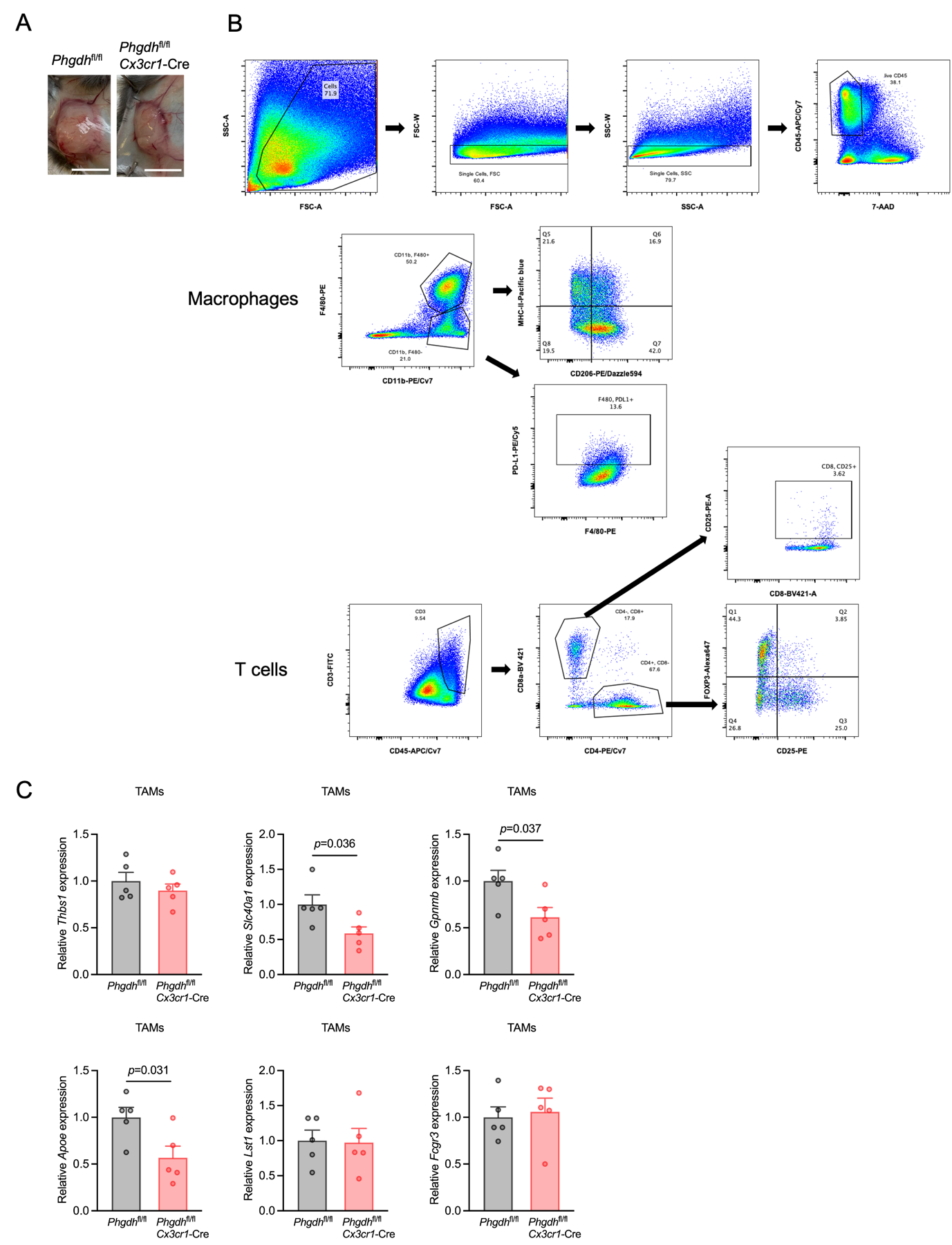


Figure S4


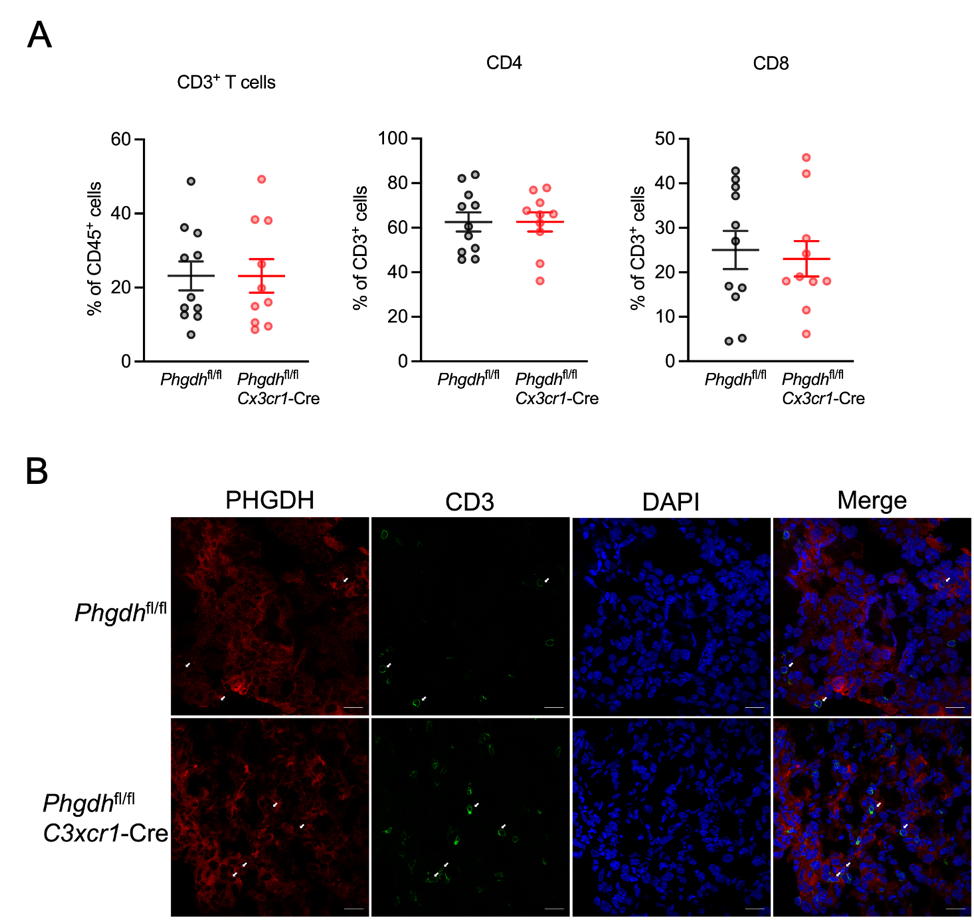


Figure S5


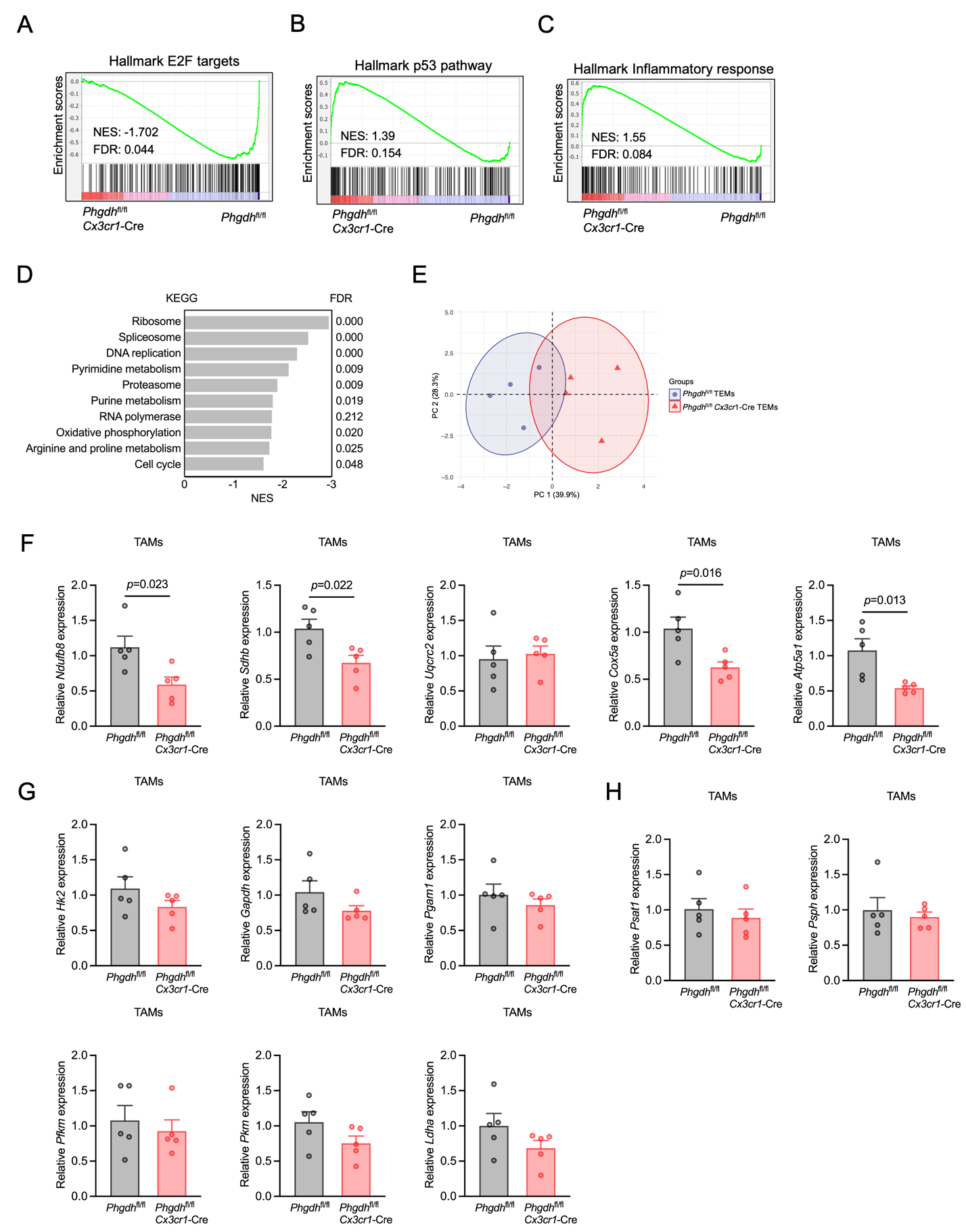


Figure S6


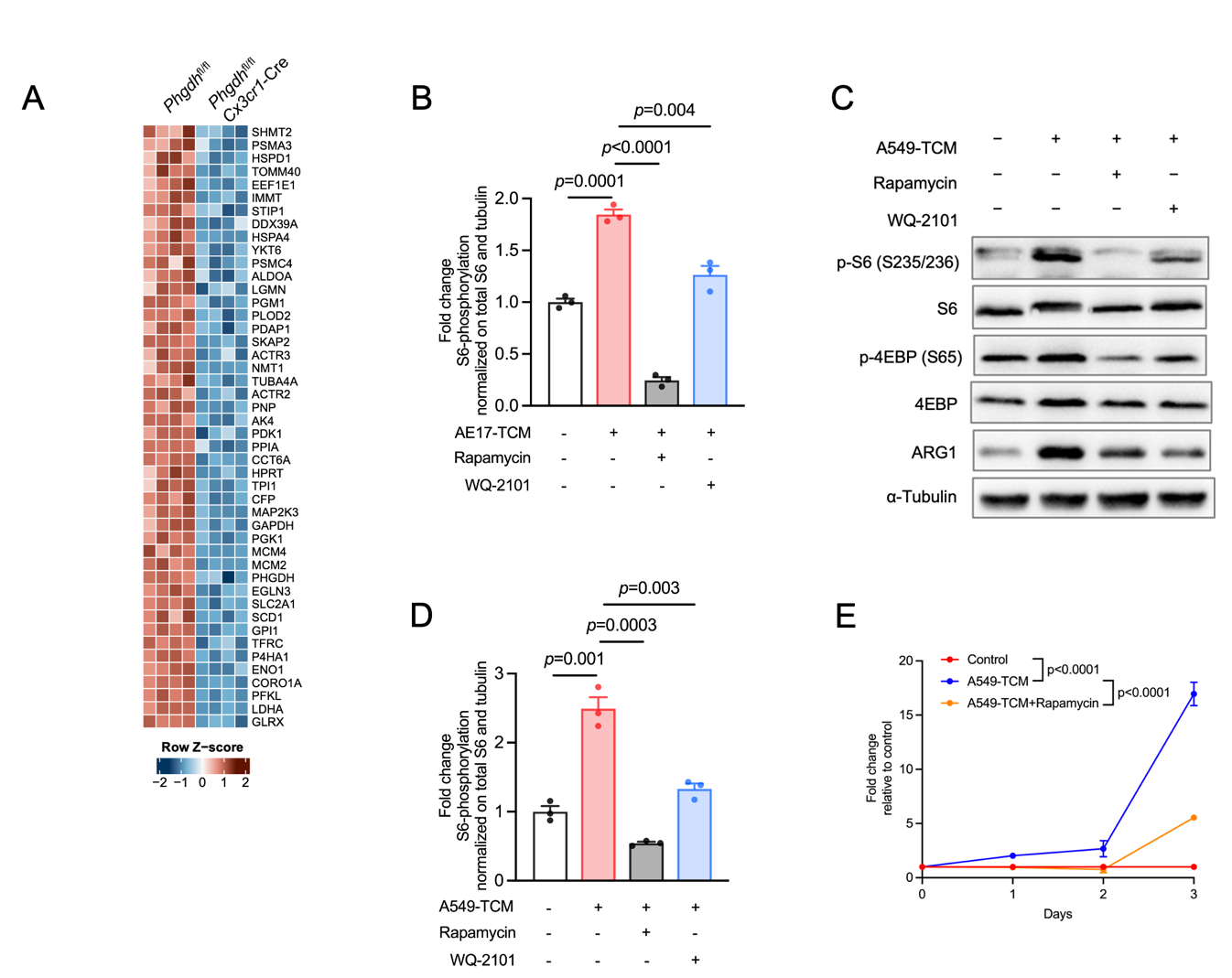


Figure S7


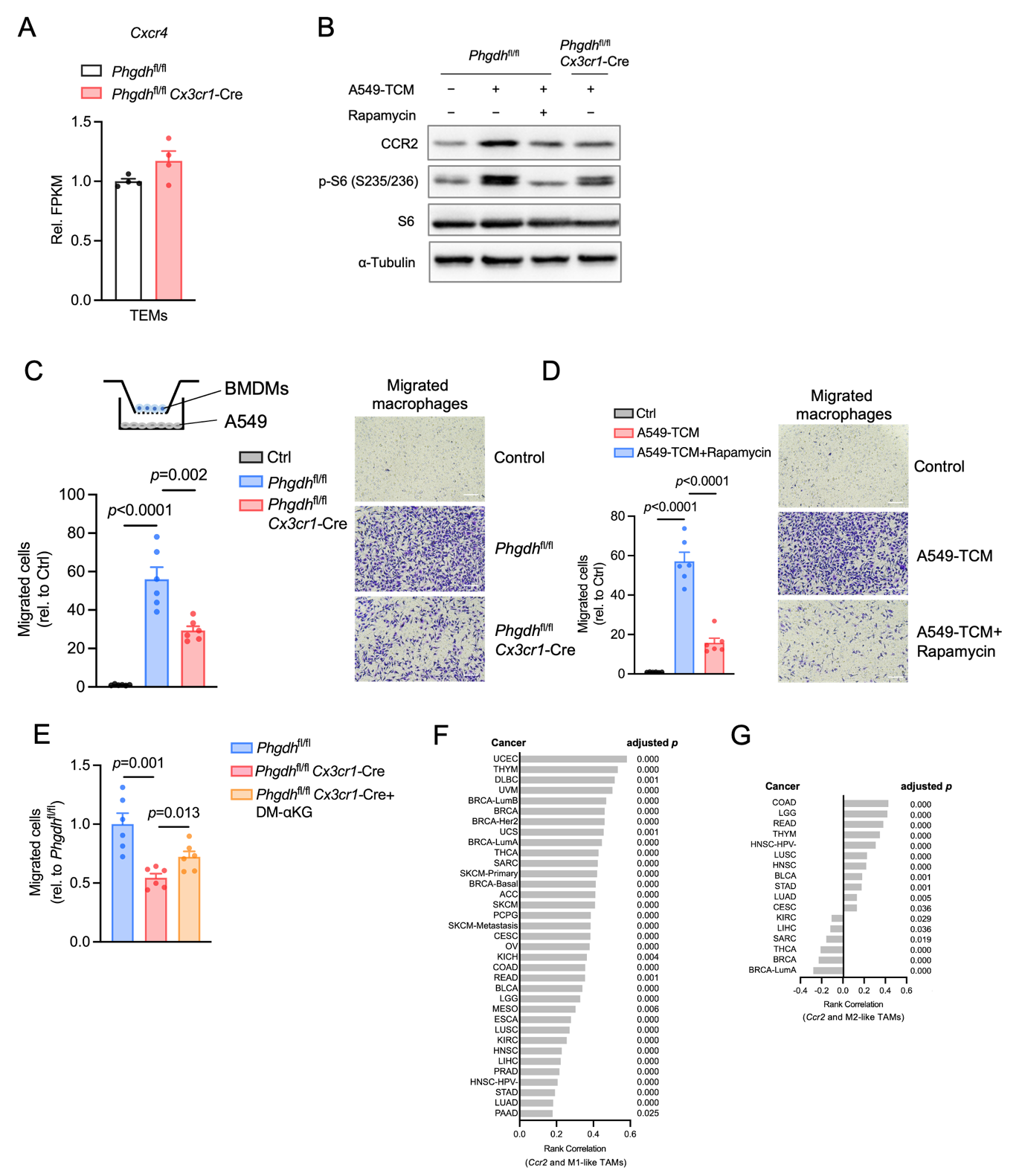

Supplement: Supplementary file 3 — Supplemental material [file 41423_2024_1134_MOESM3_ESM.docx]
