# Supplementary material for: Targeting PHGDH reverses the immunosuppressive phenotype of tumor-associated macrophages through α-ketoglutarate and mTORC1 signaling
Source: Cell Mol Immunol. 2024 Feb 27;21(5):448–65. doi: 10.1038/s41423-024-01134-0 (PMC11061172; doi:10.1038/s41423-024-01134-0)

Figure 1E

PHGDH
(57 kDa)

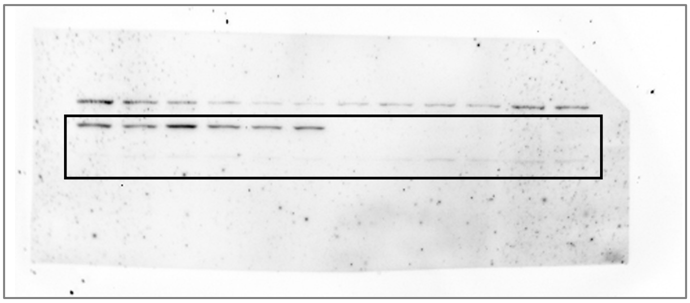

α-Tubulin
(50 kDa)

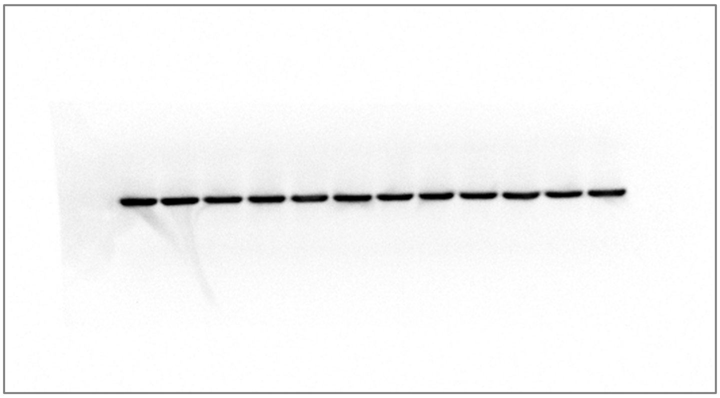

Figure 2A

ARG1
(60 kDa)

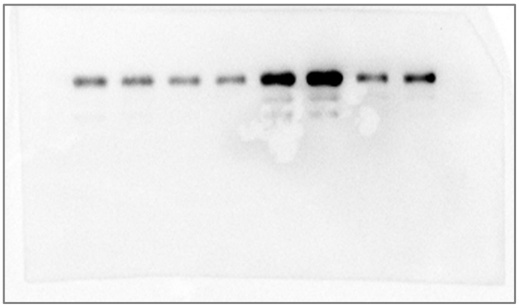

PHGDH
(57 kDa)

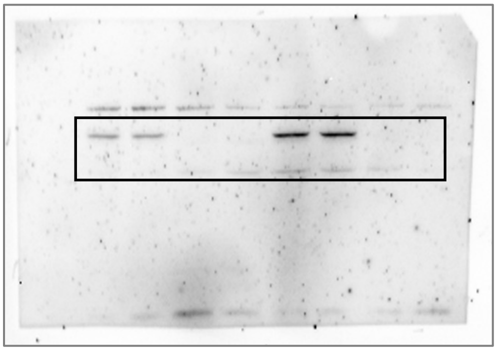

α-Tubulin
(50 kDa)

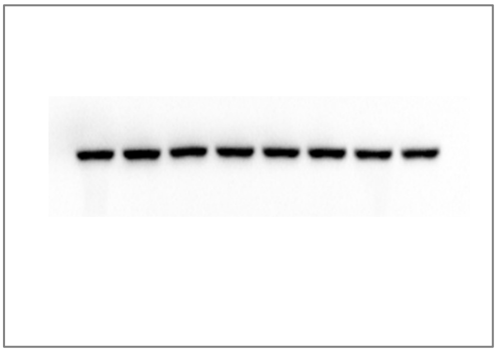

Figure 2B

ARG1

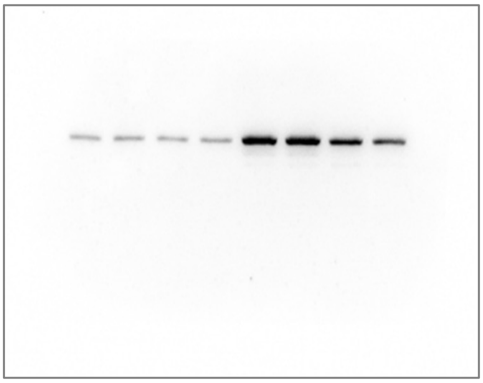

PHGDH

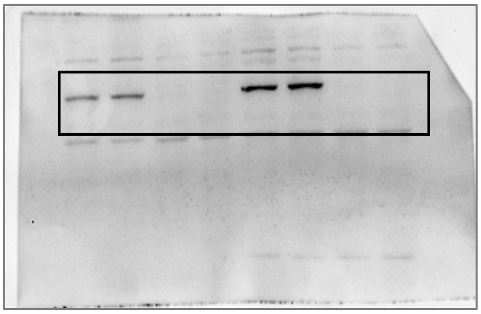

α-Tubulin

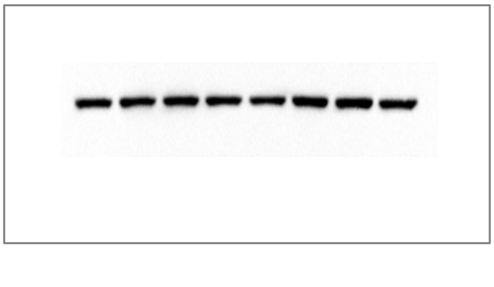

Figure 2J

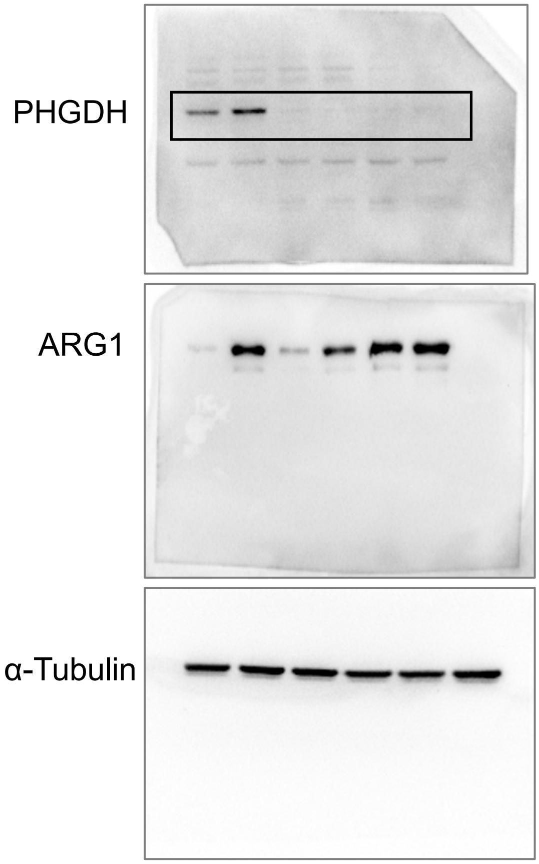

Figure 5I

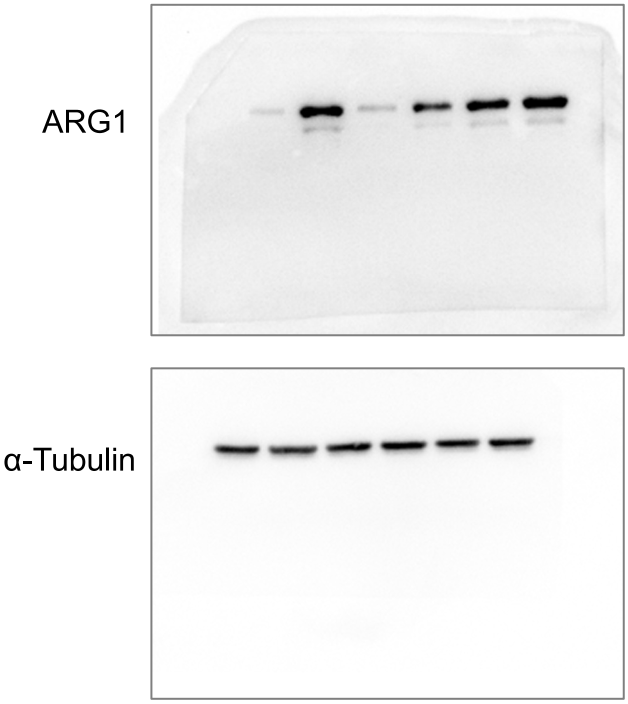

Figure 6B

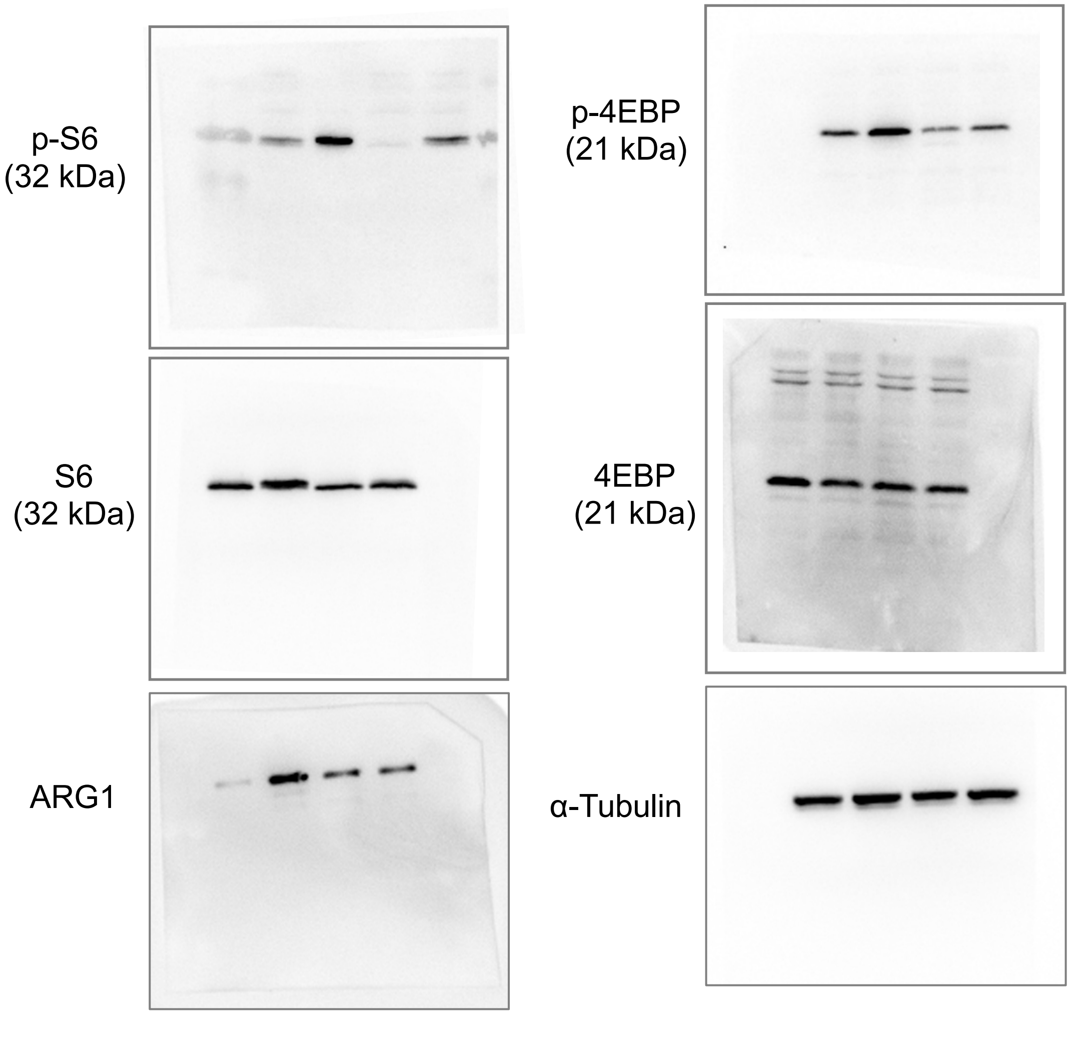

Figure 6E

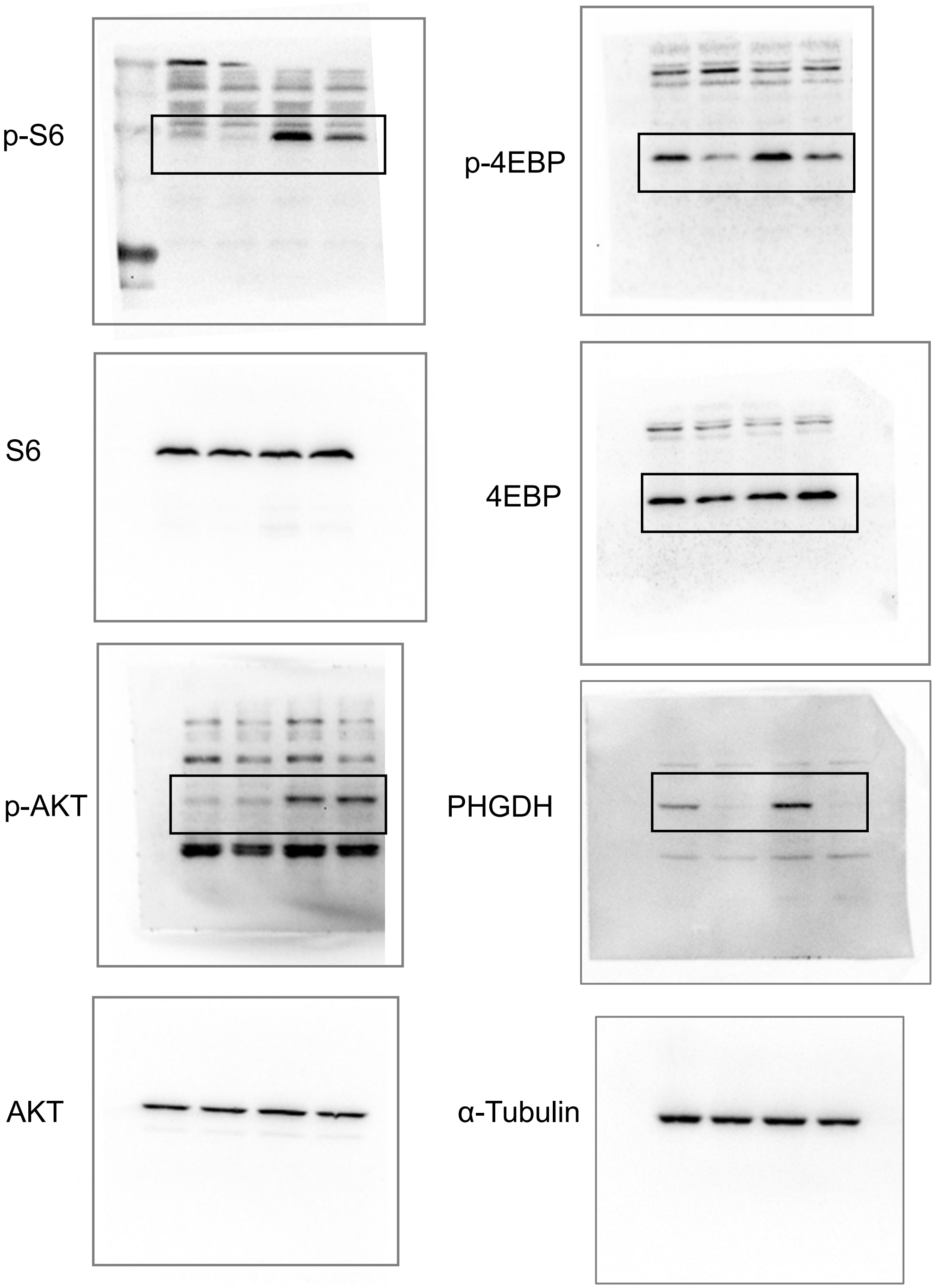

Figure 6G

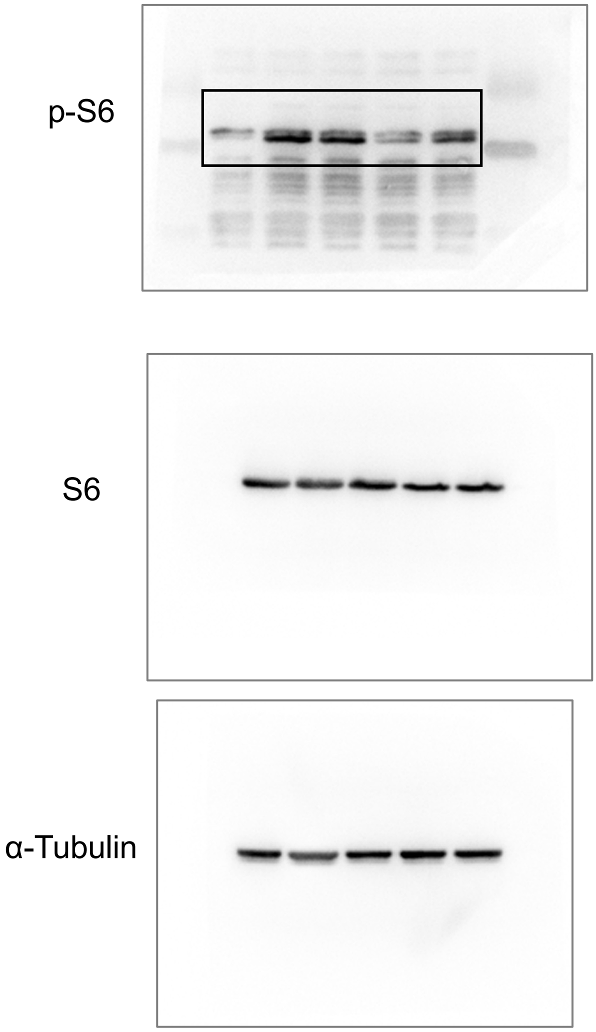

Figure 7B

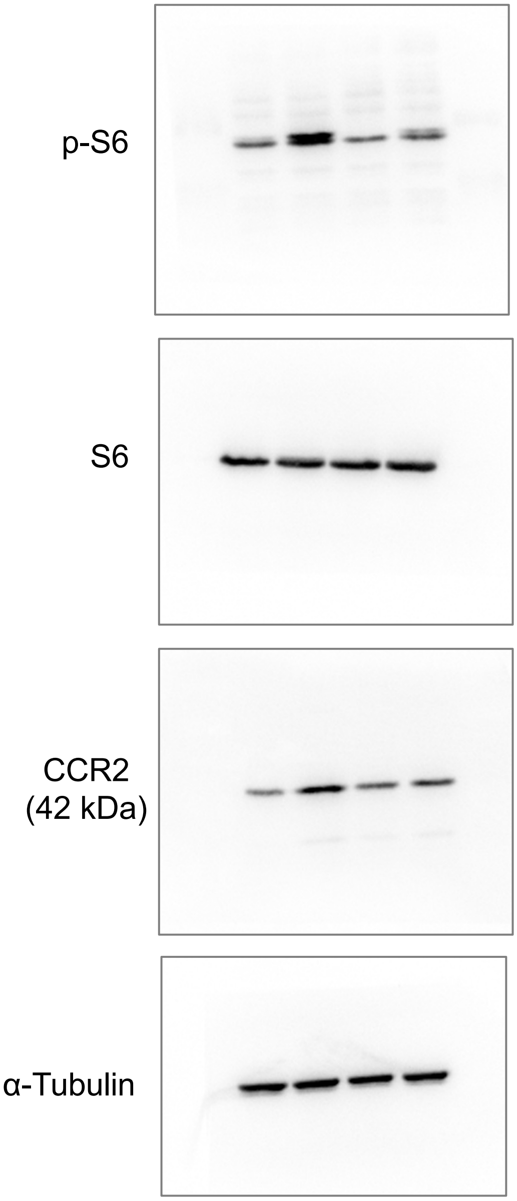

Figure S1B

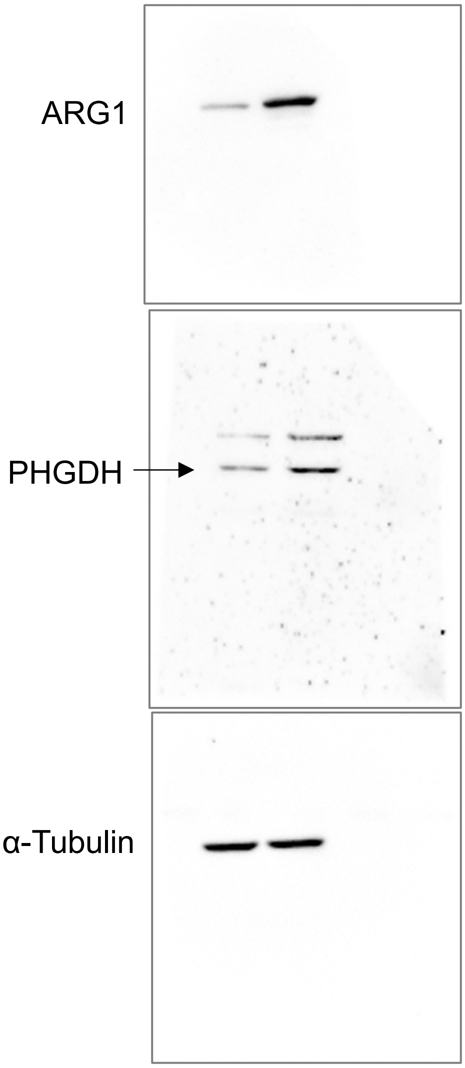

Figure S2A

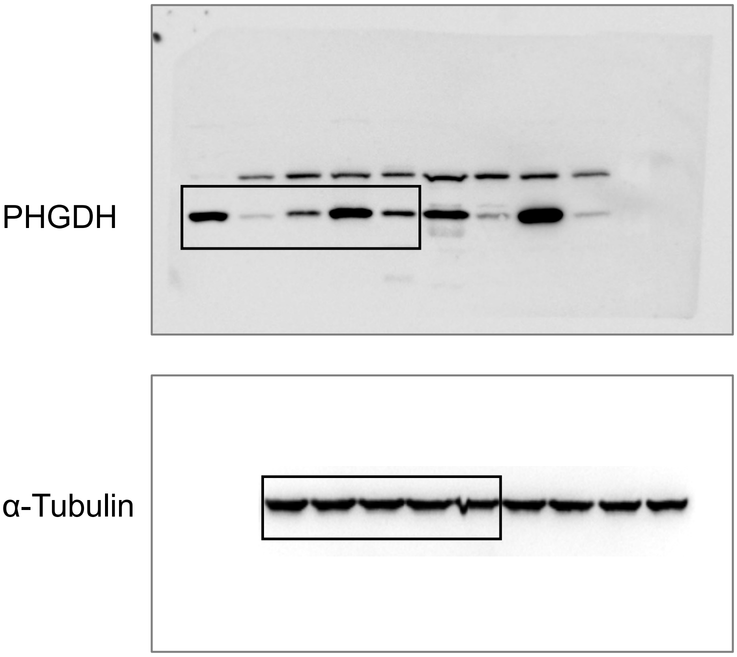

Figure S2B

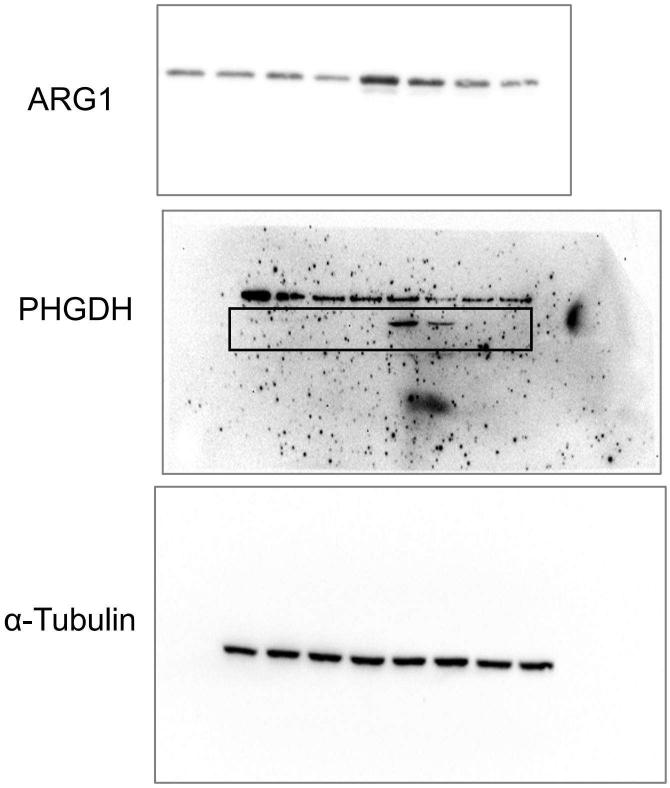

Figure S2C

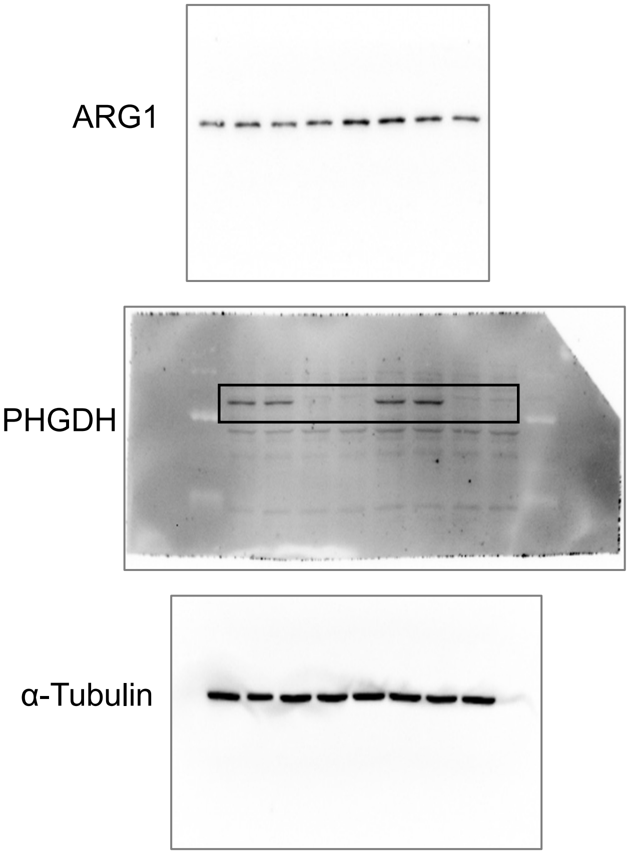

Figure S2G

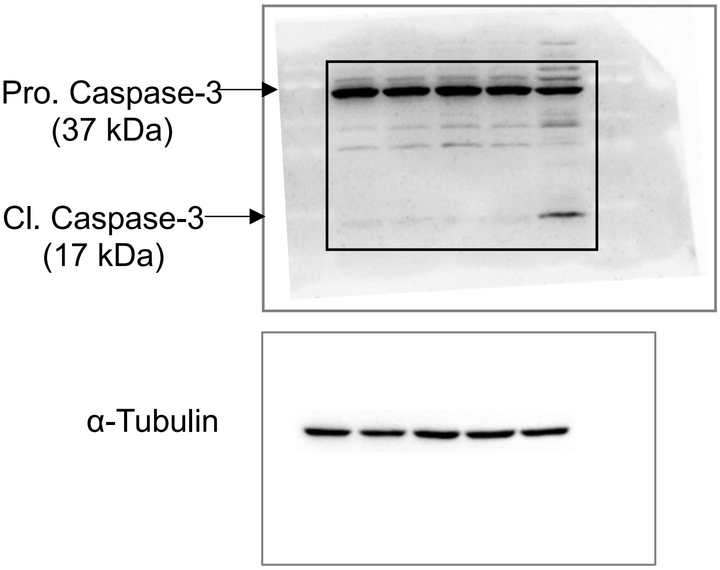

Figure S5B

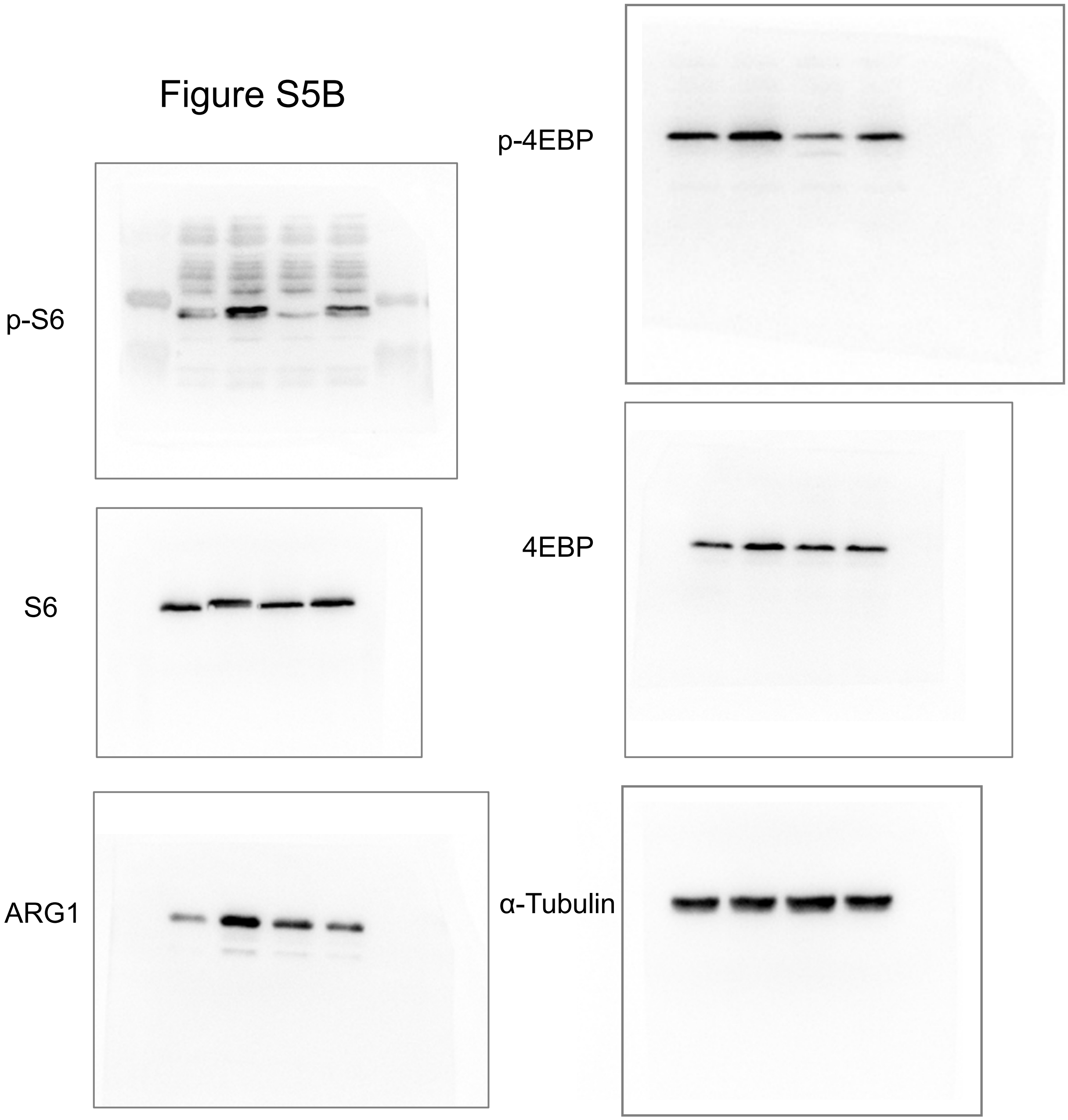

Figure S6B

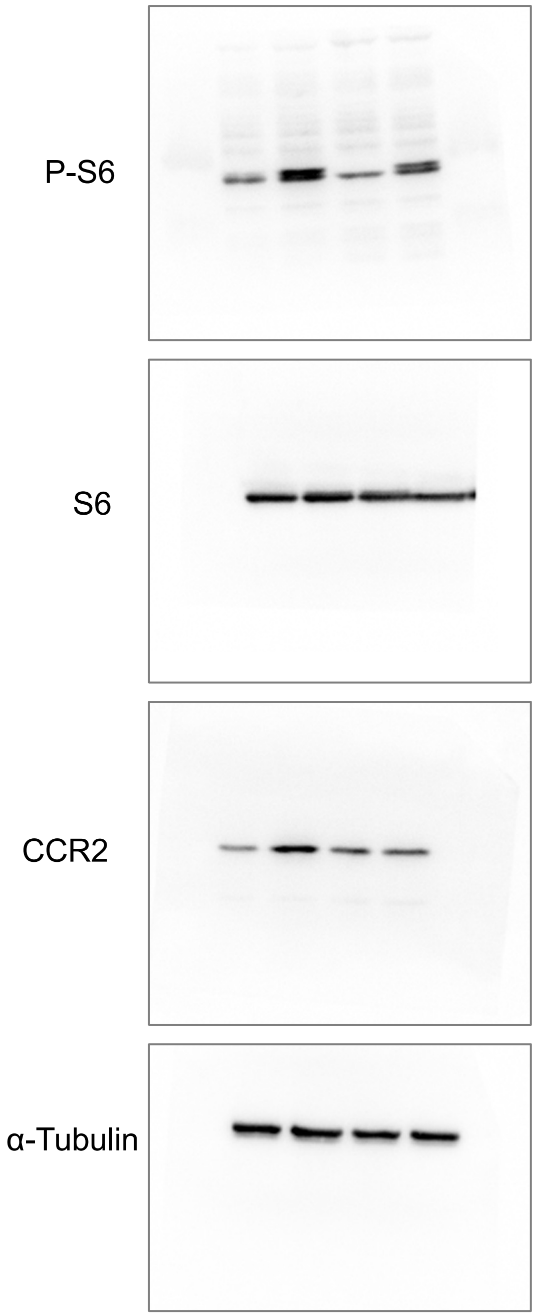

Supplement: Supplementary file 4 — Uncropped gels [file 41423_2024_1134_MOESM4_ESM.pdf]
